# Risk prediction of late-onset Alzheimer’s disease implies an oligogenic architecture

**DOI:** 10.1038/s41467-020-18534-1

**Published:** 2020-09-23

**Authors:** Qian Zhang, Julia Sidorenko, Baptiste Couvy-Duchesne, Riccardo E. Marioni, Margaret J. Wright, Alison M. Goate, Edoardo Marcora, Kuan-lin Huang, Tenielle Porter, Simon M. Laws, Australian Imaging Biomarkers and Lifestyle (AIBL) Study, Colin L. Masters, Ashley I. Bush, Christopher Fowler, David Darby, Kelly Pertile, Carolina Restrepo, Blaine Roberts, Jo Robertson, Rebecca Rumble, Tim Ryan, Steven Collins, Christine Thai, Brett Trounson, Kate Lennon, Qiao-Xin Li, Fernanda Yevenes Ugarte, Irene Volitakis, Michael Vovos, Rob Williams, Jenalle Baker, Alyce Russell, Madeline Peretti, Lidija Milicic, Lucy Lim, Mark Rodrigues, Kevin Taddei, Tania Taddei, Eugene Hone, Florence Lim, Shane Fernandez, Stephanie Rainey-Smith, Steve Pedrini, Ralph Martins, James Doecke, Pierrick Bourgeat, Jurgen Fripp, Simon Gibson, Hugo Leroux, David Hanson, Vincent Dore, Ping Zhang, Samantha Burnham, Christopher C. Rowe, Victor L. Villemagne, Paul Yates, Sveltana Bozin Pejoska, Gareth Jones, David Ames, Elizabeth Cyarto, Nicola Lautenschlager, Kevin Barnham, Lesley Cheng, Andy Hill, Neil Killeen, Paul Maruff, Brendan Silbert, Belinda Brown, Harmid Sohrabi, Greg Savage, Michael Vacher, Perminder S. Sachdev, Karen A. Mather, Nicola J. Armstrong, Anbupalam Thalamuthu, Henry Brodaty, Loic Yengo, Jian Yang, Naomi R. Wray, Allan F. McRae, Peter M. Visscher

**Affiliations:** 1 Institute for Molecular Bioscience, The University of Queensland, Brisbane, QLD 4072 Australia; 2 Centre for Genomic and Experimental Medicine, Institute of Genetics and Molecular Medicine, University of Edinburgh, Edinburgh, EH4 2XU UK; 3 Queensland Brain Institute, The University of Queensland, St Lucia, QLD 4072 Australia; 4 Centre for Advanced Imaging, The University of Queensland, St Lucia, QLD 4072 Australia; 5 Department of Neuroscience, Icahn School of Medicine at Mount Sinai, New York, NY 10029 USA; 6 Department of Genetics and Genomic Sciences, Icahn School of Medicine at Mount Sinai, New York, NY 10029 USA; 7 Collaborative Genomics Group, Centre of Excellence for Alzheimer’s Disease Research and Care, School of Medical and Health Sciences, Edith Cowan University, Joondalup, WA Australia; 8 School of Pharmacy and Biomedical Sciences, Faculty of Health Sciences, Curtin Health Innovation Research Institute, Curtin University, Bentley, WA Australia; 9 Centre for Healthy Brain Ageing, School of Psychiatry, University of New South Wales, Sydney, NSW Australia; 10 Neuropsychiatric Institute, Prince of Wales Hospital, Sydney, NSW Australia; 11 Neuroscience Research Australia, Sydney, NSW Australia; 12 Department of Mathematics and Statistics, Murdoch University, Perth, WA Australia; 13 Dementia Centre for Research Collaboration, University of New South Wales, Sydney, NSW Australia; 14 The Florey Institute, The University of Melbourne, Parkville, VIC 3052 Australia; 15 School of Medical and Health Sciences, Edith Cowan University, Joondalup, WA Australia; 16 CSIRO, Herston, QLD 4029 Australia; 17 CSIRO, Melbourne, VIC Australia; 18 Department of Molecular Imaging, Austin Health, Heidelberg, VIC 3084 Australia; 19 National Ageing Research Institute, Parkville, VIC 3052 Australia; 20 Bio21 Institute of Molecular Science and Biotechnology, The University of Melbourne, Parkville, VIC 3052 Australia; 21 The University of Melbourne, Parkville, VIC 3052 Australia; 22 Cogstate Ltd., Melbourne, VIC Australia; 23 St. Vincent Hospital, Fitzroy, VIC 3065 Australia; 24 Department of Exercise Science, College of Science, Health, Engineering and Education, Murdoch University, Murdoch, WA Australia; 25 Department of Psychology, Macquarie University, Sydney, NSW 2109 Australia; 26 CSIRO, Floreat, WA Australia

**Keywords:** Genome informatics, Genetics, Genome-wide association studies, Alzheimer's disease

## Abstract

Genetic association studies have identified 44 common genome-wide significant risk loci for late-onset Alzheimer’s disease (LOAD). However, LOAD genetic architecture and prediction are unclear. Here we estimate the optimal *P*-threshold (*P*_optimal_) of a genetic risk score (GRS) for prediction of LOAD in three independent datasets comprising 676 cases and 35,675 family history proxy cases. We show that the discriminative ability of GRS in LOAD prediction is maximised when selecting a small number of SNPs. Both simulation results and direct estimation indicate that the number of causal common SNPs for LOAD may be less than 100, suggesting LOAD is more oligogenic than polygenic. The best GRS explains approximately 75% of SNP-heritability, and individuals in the top decile of GRS have ten-fold increased odds when compared to those in the bottom decile. In addition, 14 variants are identified that contribute to both LOAD risk and age at onset of LOAD.

## Abstract (web summary)

Despite the identification of genetic risk loci for late-onset Alzheimer’s disease (LOAD), the genetic architecture and prediction remains unclear. Here, the authors use genetic risk scores for prediction of LOAD across three datasets and show evidence suggesting oligogenic variant architecture for this disease.

## Introduction

Alzheimer’s disease (AD) is the most common form of dementia. The majority (~90–95%) of AD cases are sporadic and occur after 65 years of age (late-onset Alzheimer’s disease, LOAD)^1^. The reported heritability of LOAD liability is 58.0% (95% CI 19.0–87.0%) from twin studies^2^, and its estimated common single nucleotide polymorphism (SNP) based heritability on the liability scale ($h_{\rm{SNP(l)}}^2$) ranges from 0.13 to 0.33^3–5^. *APOE* alleles (*ɛ*2, *ɛ*3 and *ɛ*4, determined by two coding variants, rs7412 and rs429358 from chromosome 19), especially *APOE* *ɛ*4, explain around a quarter of the total heritability^6,7^, and can be regarded as a proxy monogenic mutation.

In addition to *APOE* alleles, genome-wide association studies (GWASs) have identified over 40 LOAD-associated risk loci^8–15^. Similar to other brain-related diseases (e.g., schizophrenia^16,17^, major depression^18^ and Parkinson’s disease^19^), LOAD has been described as polygenic^20^. A genetic risk score (GRS) derived from 13,959 cases and 35,600 controls based on a large number of SNPs (i.e., SNPs with *P*_GWAS_ ≤ 0.5) was reported to have better prediction accuracy than using SNPs selected with a more stringent *P*_GWAS_. However, a recent study^14^ with 24,087 AD cases, 47,793 family history proxy cases, 55,058 controls and 328,320 proxy controls showed that the optimal *P*-threshold (*P*_optimal_) for prediction was achieved with a stringent threshold of ~10^−5^, which implies that using more SNPs at lower stringency does not improve prediction accuracy. The *P*_optimal_ of GRS on diseases (e.g., schizophrenia) was previously reported to be related to the discovery sample size^21^. Nevertheless, it was observed that the best fitting *P-*value for GRS prediction of schizophrenia changed little from 0.2 with 2615 cases and 3338 controls to 0.1 with 32,838 cases and 44,357 controls^16^. The reasons for this inconsistency in *P*_optimal_ for LOAD (from 0.5 to ~10^−5^) across studies is unclear, in particular whether it may be solely due to the increase of discovery sample size. These conflicting reports on the number of common risk variants associated with LOAD led us to investigate the genetic architecture of the disease, and to compare the prediction accuracy between a multiple SNP genetic predictor of LOAD (including or excluding *APOE*) versus *APOE* alone.

For LOAD, age at onset (AAO) is also heritable. Its heritability is reported to be 0.42 (s.e. = 0.04)^22^ and can be predicted genetically using a genetic hazard score (GHS)^23^. The effect size of each SNP in GHS is usually estimated based on Cox proportional hazards regression (survival analysis)^24^. Previous studies have identified four genomic regions (*APOE*, *BIN1*, *MS4A* and *PICALM*) with SNPs genome-wide significantly (*P* < 5 × 10^−8^) associated with LOAD AAO, all of these being LOAD risk loci^13,25–28^. A direct comparison of LOAD risk and AAO on the same data may provide new insight into the genetics of LOAD.

In the present study, we investigate the prediction pattern of GRS to estimate the optimal *P-*value cut-off, and thereby quantify the genetic architecture of LOAD. To ensure the robustness of our results, we use four sets of (overlapping) GWAS summary statistics to calculate the GRS (with or without SNPs from chromosome 19) and examine their prediction patterns in three independent datasets (out-of-sample prediction). The results suggest that LOAD is oligogenic compared to other disorders of the brain, since only a small number of common SNPs are conditionally associated with LOAD. Furthermore, we compare the prediction performance of GRS against *APOE* and find that individuals in the upper decile of GRS have higher disease risk than those who are *APOE* *ɛ*4 heterozygous carriers. Finally, risk of LOAD and AAO of LOAD are found to be genetically similar.

## Results

### Current GWAS summary statistics on late-onset Alzheimer’s disease

**Fig. 1:**
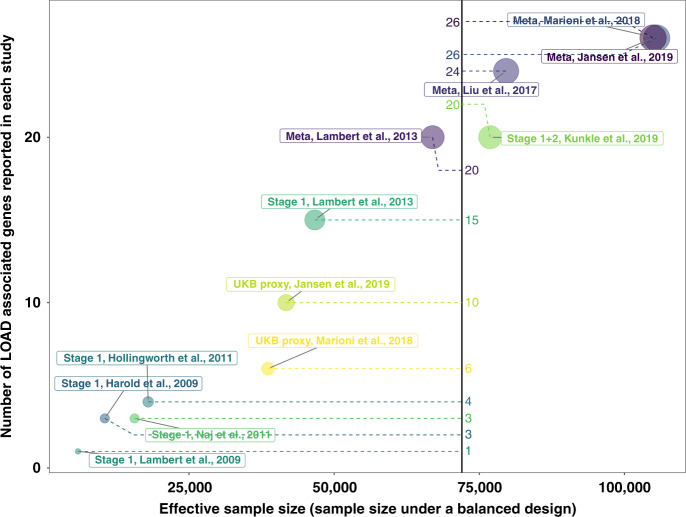
The relationship between sample size and number of identified genes. Sample size is calculated as the total number of cases and controls under a balanced design (50% cases and 50% controls). Genes associated with LOAD were collected from different studies. They are the closest genes to SNPs (minor allele frequency >0.01) genome-wide significantly (*P*< 5 × 10^−8^) associated with LOAD. “Stage 1” means summary statistics based on the samples from stage 1 in that study and “Meta” means summary statistics from the meta-analysis from that study.

To date, eight studies^8–15^ have reported a total of 44 common loci (minor allele frequency >0.01) that are associated with LOAD at a genome-wide significant level (*P* < 5 × 10^−8^) (Supplementary Fig. 1). As expected, the number of reported loci increased with effective sample size (Fig. 1) (Supplementary methods).

We collected four sets of GWAS summary statistics from the public domain to calculate GRS^12–14^. They are based on samples from stage 1 in Lambert et al.^12^, samples from UK Biobank (UKB) parents (a meta-analysis between GWASs on maternal and paternal LOAD), a meta-analysis between summary statistics from Lambert et al.^12^ and UKB parents in Marioni et al.^13^, and a recent meta-analysis from Jansen et al.^14^. These summary statistics are from samples with partial overlap and some of them are independent (i.e., samples from Lambert et al.^12^ and UKB parents). Genetic correlations between these summary statistics estimated by LDscore regression (LDSC)^29^ were all close to unity (Supplementary Table 1). Among them, two estimates (genetic correlations between Lambert et al. (stage 1)^12^/Marioni et al. (UKB)^13^ and Marioni et al. (meta)^13^) were significantly (*P* < 0.05) different from one (Supplementary Table 1). This discrepancy was not expected since they were all GWAS results on the same trait and had overlapping samples. LDSC assumes that the effect sizes of SNPs follow a normal distribution, we therefore removed all SNPs from chromosome 19 to avoid the potential effect of *APOE* when estimating the genetic correlation. We also re-calculated the sample size for each SNP based on the standard error of its effect size (“Methods”). We used the flag “--intercept-gencov” to constrain the intercept by our calculated value while computing the genetic correlation. We found that the estimated genetic correlation between Marioni et al. (UKB)^13^ and Marioni et al. (meta)^13^ was 1.06 (s.e. = 0.11), and the genetic correlation between Lambert et al. (stage 1)^12^ and Marioni et al. (meta)^13^ was 1.14 (s.e. = 0.11), both not significantly (*P* > 0.05) different from unity. We noted that the sample size and therefore the weights used in the meta-analysis of Jansen et al.^14^ were not optimal and show that the effective sample size (sample size under balanced design) should be used (Supplementary methods).

### Genetic risk score in late-onset Alzheimer’s disease

**Fig. 2:**
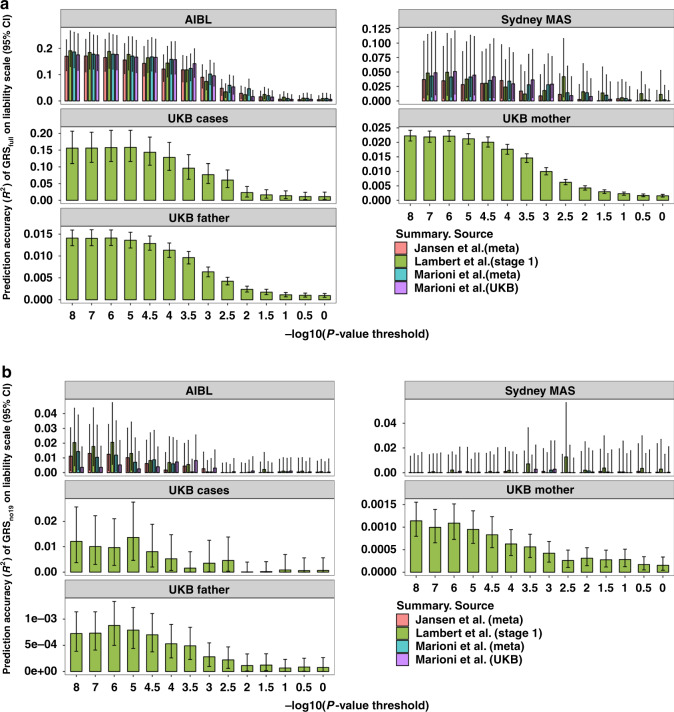
The prediction performance of genetic risk score (GRS) in different datasets. **a** The prediction accuracy of GRS_full_ based on SNPs selected using different *P-*value thresholds. GRS_full_ was calculated based on 1,056,154 HapMap3 SNPs and two *APOE* SNPs. **b** The prediction accuracy of GRS_no19_ based on SNPs selected using different *P-*value thresholds. GRS_no19_ is calculated based on HapMap3 SNPs excluding SNPs from chromosome 19, to avoid contamination with *APOE*. Prediction results on samples from UKB cases and UKB parents are based on summary statistics from Lambert et al.^12^ (stage 1) only. The error bars represent 95% confidence interval, and the confidence interval was calculated based on 1000 bootstrap replications^59^.

We used 1,056,156 SNPs (1,056,154 HapMap3 SNPs and two *APOE* SNPs: rs429358 and rs7412) shared between all four sets of summary statistics to calculate the GRS (GRS_full_). We retained HapMap3 SNPs in our study since they are common (minor allele frequency >0.01), well-imputed and available across all GWASs. For each set of summary statistics, we chose different *P-*value thresholds (1 × 10^−8^, 1 × 10^−7^, 1 × 10^−6^, 1 × 10^−5^, 3 × 10^−5^, 1 × 10^−4^, 3 × 10^−4^, 1 × 10^−3^, 3 × 10^−3^, 0.01, 0.03, 0.1, 0.3, 1) and performed LD clumping (*R*^2^ = 0.01, window size = 1 Mbp) to select near-independent SNPs using PLINK^30^. Based on the selected SNPs, we calculated the weighted sum of the SNP dosage and used it as the GRS for each individual^21^. We evaluated the performance of GRS_full_ using samples from the Australian Imaging, Biomarker & Lifestyle Study (AIBL, 216 cases and 631 controls), the Sydney Memory and Ageing study (Sydney MAS, 77 cases and 588 controls) and the UKB (383 cases and 1915 controls) (Table 1). We found that the prediction accuracy (*R*^2^) on the liability scale (Fig. 2a) (“Methods”) increased when lowering the *P-*value threshold. Since the prediction pattern could be affected by the SNPs with major effects (e.g., *APOE* *ɛ*4 and *ɛ*2) (Supplementary Fig. 2) (“Methods”), we removed SNPs from chromosome 19 and re-calculated the GRS based on the remaining 1,037,804 SNPs (termed GRS_no19_). Although the *R*^2^ reduced compared to that from GRS_full_, the optimal *P-*value threshold remained small (Fig. 2b). The *P-*value thresholds that maximised out-of-sample prediction (*R*^2^) in AIBL were 1 × 10^−8^ (Lambert et al., stage 1^12^), 1 × 10^−7^ (Jansen et al., meta^14^), 1 × 10^−8^ (Marioni et al., meta^13^) and 3 × 10^−4^ (Marioni et al., UKB^13^). Samples from UKB were only evaluated based on summary statistics from Lambert et al. (stage 1)^12^ to avoid the sample overlap. Results based on Sydney MAS were highly variable (Fig. 2b) since the number of cases is small, yielding limited power compared to the other two cohorts (Fig. 2b). We found that the odds ratio between individuals in the top 50% of GRS_no19_ and those in the bottom 50% (Supplementary Fig. 3) also increased with a decrease in *P-*value threshold. We further explored the GRS_no19_ prediction performance of Lambert et al. (stage 1)^12^ on the UKB parental LOAD (Table 1). Although the prediction accuracy is small, its pattern is consistent with that from other cohorts (Fig. 2b). Furthermore, we used less stringent *R*^2^ (0.2) to perform LD clumping so that more SNPs could be included in GRS_no19_. We found no improvement in prediction accuracy or change in the pattern (Supplementary Fig. 4). In addition, we estimated the optimal fraction of causal SNPs for prediction using LDpred^31^ (on SNPs outside of chromosome 19) (“Methods”) (Supplementary Fig. 5), and found the optimal proportion of SNPs was lower than 0.3% in most situations. Given the LD between SNPs, the number of effective independent markers would be even lower.

**Table 1:** Description of late-onset Alzheimer’s disease cases and controls from different cohorts.

	*N*_(proxy) case_	*N*_(proxy) control_	Age^a^ (sd) _(proxy) case_	Age^a^ (sd)_(proxy) control_	Female
AIBL^b^	216	631	77.6 (7.6)	72.2 (6.4)	45.3%
Sydney MAS^c^	77	588	86.8 (4.6)	84.7 (4.5)	55.6%
UKB	383	1915	64.4 (4.5)	64.5 (2.6)	53.0%
UKB mother	22,557	231,767	83.7 (6.8)	78.1 (8.4)	100%
UKB father	13,118	241,206	81.8 (6.9)	76.2 (8.1)	0%

^a^For the UKB mother and father samples, age of parental case was used as a proxy for age at onset.

^b^The Australian Imaging Biomarkers and Lifestyle Study.

^c^The Sydney Memory and Ageing Study.

The highest prediction accuracy of GRS_full_ (based on 22 SNPs, Supplementary Table 2) was 19.1% (95% bootstrap CI 13.1–26.9%, 1000 replications) of variance explained on the liability scale (“Methods”), with *APOE* (rs429358 and rs7412) contributing the majority (17.4%, 95% bootstrap CI 11.3–25.0%, 1000 replications). We compared this prediction accuracy with the transformed common SNP-based heritability on the liability scale ($h_{\rm{SNP(l)}}^2$) reported in previous studies (ranges from 8.9 to 31.2% across studies)^3–5^ (Supplementary Table 3 and Supplementary Fig. 6) (“Methods”). The SNP-heritability was estimated by different methods and our simulations (“Methods”) suggested that when most of the SNP-based heritability was explained by a single variant, the estimated value from LDSC was lower than the simulated heritability, but the result from genome-based restricted maximum likelihood (GREML) was unbiased (Supplementary Fig. 7). Therefore, only $h_{\rm{SNP(l)}}^2$ based on GREML is considered here. We found that the prediction accuracy achieved could account for around three quarters of inverse-variance weighted average of $h_{\rm{SNP(l)}}^2$ (26.2%, 95% CI 22.7–29.7%), suggesting that the best GRS_full_ could explain most of the SNP-heritability. Besides, the best GRS_full_ accounts for one-third of the reported total heritability (58.0%, 95% CI 19.0–87.0%) from twin studies^2^ (Supplementary Fig. 8). However, the differences between the prediction accuracy of *APOE*, GRS_full_, $h_{\rm{SNP(l)}}^2$, and total heritability are not statistically significant (*P* > 0.05).

### Genetic architecture and optimal threshold in GRS

**Fig. 3:**
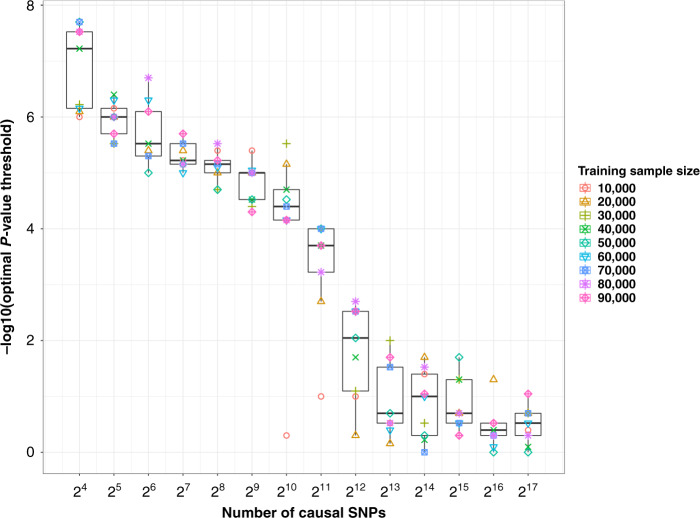
The relationship between optimal GRS *P-*value threshold and number of causal SNPs. Causal SNPs were selected from 1,037,804 HapMap3 SNPs. For each scenario, we generated a phenotype of 100,000 individuals based on a specified number of causal SNPs (e.g., 128) with heritability 0.09. We randomly selected 10,000 individuals as the test set. Based on the unselected individuals, we randomly chose 10,000, 20,000, 30,000, 40,000, 50,000, 60,000, 70,000, 80,000 and 90,000 individuals separately as training sets and used them to perform GWAS. We examined the performance of genetic risk score (based on LD clumping with 80 separate *P-*value thresholds) on the test set (*N*_test_ = 10,000) and selected the optimal *P-*value threshold. Box plot shows the median (centre line), the interquartile range (box) and whiskers (±1.5 times interquartile range).

The prediction pattern of GRS on LOAD is different from that of polygenic traits like BMI^32^, height^32^, schizophrenia^16^ and major depression^18^. Our simulation study suggests that this difference is related to their distinct genetic architectures, and that LOAD is much less polygenic compared to these other complex traits. In our simulations, we randomly selected 100,000 unrelated individuals from the UKB and simulated traits with an SNP-heritability of 9% (close to the reported SNP-heritability of LOAD excluding the effect of *APOE*), varying the number of causal variants (“Methods”)^21^. We selected 10,000 individuals as a (hold–out) test set and chose different number of individuals (from 10,000 to 90,000) as a training set. We ran GWAS on the training set and examined the prediction pattern of the GRS on the test set. We observed an increase in the optimal *P-*value threshold of GRS as the number of causal SNPs increases (from 16 to 131,072) (Fig. 3 and Supplementary Fig. 9). The pattern of GRS on LOAD was consistent with simulations on fewer than 256 causal SNPs (*P*_optimal_ < 1 × 10^−5^). In addition, we used a recently developed Bayesian regression method (SBayesR^33^) that estimates the number of SNPs with non-zero effect size from GWAS summary statistics. We only used the Marioni et al. (meta)^13^ summary statistics, since these are based on the largest effective sample size (“Methods”). We estimated the number of SNPs with non-zero effects on LOAD to be 99 (s.e. = 6), which represents only ~0.01% of HapMap3 SNPs. This number decreased to 56 (s.e. = 6), if SNPs from chromosome 19 are removed before the analysis. For context, these estimates are much lower than those of other common diseases such as Parkinson’s disease (33,728, s.e. = 11,968), schizophrenia (184,879, s.e. = 25,250) and major depression (172,735, s.e. = 43,219) (“Methods”).

### Comparison of prediction performance between GRS and *APOE*

**Fig. 4:**
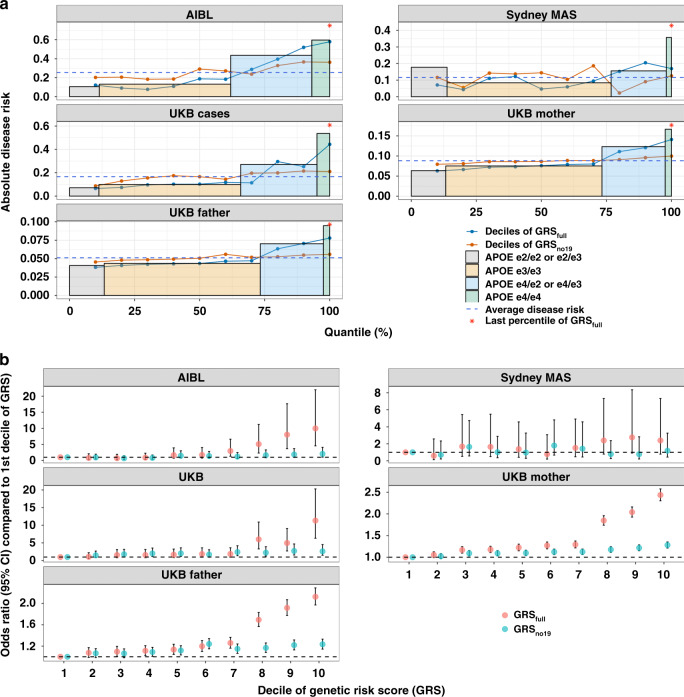
The comparison of LOAD prediction performance between GRS and *APOE*. **a** The disease risk of late-onset Alzheimer’s disease of individuals in different deciles of GRS (both GRS_full_ and GRS_no19_), last percentile of GRS_full_ and in individuals with *APOE* *ɛ*2/*ɛ*2 or *ɛ*2/*ɛ*3, *APOE* *ɛ*3 homozygotes (*ɛ*3/*ɛ*3), *APOE* *ɛ*4 heterozygotes (*ɛ*4/*ɛ*3 or *ɛ*4/*ɛ*2) and *APOE* *ɛ*4 homozygotes (*ɛ*4/*ɛ*4). Samples from AIBL, Sydney MAS, UKB cases, UKB mother and UKB father were examined. **b** Odds ratio between individuals in the other deciles and first decile of GRS. GRS_full_ was calculated based on 1,056,154 HapMap3 SNPs and two *APOE* SNPs. GRS_no19_ was calculated based on HapMap3 SNPs but excluding SNPs from chromosome 19. Only independent (*R*^2^ < 0.01) SNPs with *P* < 1 × 10^−8^ were used to calculate the GRS. The error bars in **b** represent 95% confidence interval.

For coronary artery disease, GRS could identify individuals with risk equivalent to monogenic mutations^34^. Here, we compared the prediction performance of *APOE* with GRS (based on the most stringent *P-*value threshold: 1 × 10^−8^). In AIBL, individuals who are *APOE* *ɛ*4 heterozygous carriers were found to have a higher disease risk (43.6%) than those in the highest decile of a GRS_no19_ (35.7%). Using both *APOE* SNPs and variants on other chromosomes, the disease risk of individuals in the top decile of the GRS_full_ was 57.1% (Fig. 4a). The odds ratio was 10.0 (95% CI 4.5–22.0) compared to individuals in the bottom decile (Fig. 4b). This disease risk is larger than the individuals who are *APOE* *ɛ*4 heterozygous carriers (43.6%), but smaller than individuals who are homozygous for *APOE* *ɛ*4 (59.6%). Nevertheless, individuals in the last percentile of GRS_full_ have larger disease risk (75.0%) than individuals who are homozygous for *APOE* *ɛ*4. We observed the same pattern in the Sydney MAS and UKB samples (Fig. 4a). Across the different target datasets, around 1% improvement of the area under the ROC curve (AUC) could be achieved by a GRS_full_ (ranges from 57.1 to 73.2%) compared to *APOE*. Ignoring SNPs from chromosome 19, the AUC based on GRS_no19_ ranges from 51.8% (95% CI 51.4–52.3%) to 59.0% (95% CI 54.2–63.1%), all of them are significantly different (*P-*value < 0.05) from 50% (Supplementary Fig. 10).

### Genetic similarity between LOAD risk and AAO

To explore whether there are more genomic loci associated with both LOAD risk and AAO, we tried to detect new AAO loci and investigate whether they have been identified to be associated with LOAD risk. We used the parental AAO of LOAD as reported in UKB as a proxy of AAO and performed genome-wide survival analysis (GWSA) on maternal and paternal AAO of LOAD separately (“Methods”). Six independent (pairwise *R*^2^ < 0.01) genome-wide significant (*P* < 5 × 10^−8^) SNPs were identified after meta-analysing the parental AAO results (Supplementary Fig. 11a). Furthermore, we meta-analysed the UKB results with previously reported AAO GWSA summary statistics^28^, and identified 16 genomic loci with SNPs showing genome-wide significant (*P* < 5 × 10^−^^8^) association with LOAD AAO (Table 2) (Supplementary Fig. 11b). Among these, 14 loci were genome-wide significantly associated (*P* < 5 × 10^−8^) with LOAD risk, the remaining two SNPs also have *P-*values <5 × 10^−5^. The correlation between the effect sizes of the 16 SNPs on disease risk and AAO was 1.00 (s.e. = 0.02), suggesting the risk alleles of LOAD also decrease the AAO of LOAD.

**Table 2:** Genome-wide significant SNPs associated with age at onset (AAO) of late-onset Alzheimer’s disease.

CHR	BP	SNP	A1	Closest Gene^a^	BETA_AAO	P_AAO	BETA_RISK^b^	P_RISK^b^
1	207786289	rs6701713^c^	A	*CR1*	0.079	3.5E − 12	0.132	1.6E − 28
2	127891427	rs4663105^c^	A	*LOC105373605*	−0.139	1.8E − 28	−0.162	7.3E − 49
6	32573415	rs601945^c^	A	*HLA-DRB1*	0.070	4.2E − 08	0.106	1.2E − 14
6	47432637	rs9381563^c^	T	.	−0.066	1.2E − 08	−0.075	5.8E − 14
7	99990364	rs34995835^c^	T	*PILRA*	−0.066	1.3E − 10	−0.094	1.1E − 18
8	27464929	rs4236673^c^	A	*CLU*	−0.074	1.9E − 10	−0.110	1.1E − 28
11	85867875	rs10792832^c^	A	*PICALM*	−0.088	2.9E − 20	−0.124	5.1E − 36
11	121435587	rs11218343^c^	T	*SORL1*	0.171	6.1E − 12	0.213	4.8E − 17
11	60021948	rs1582763^c^	A	*MS4A*	−0.084	2.5E − 18	−0.088	1.0E − 18
14	92937293	rs4904929^c^	T	*SLC24A4*	−0.068	6.0E − 09	−0.075	1.1E − 10
15	59022615	rs442495^c^	T	*ADAM10*	0.058	5.3E − 09	0.067	5.5E − 11
17	5139808	rs58124010^c^	T	*SCIMP*	0.078	2.6E − 08	0.108	2.0E − 10
19	45412955	rs1081105^c^	A	*APOE*	−0.783	5.1E − 216	−0.941	6.5E − 199
19	1039444	rs3795065^c^	T	*ABCA7, CNN2*	−0.076	1.2E − 08	−0.077	3.7E − 10
1	161151844	rs11265563^d^	A	*B4GALT3*	0.078	8.9E − 09	0.049	4.4E − 05
4	11027619	rs4351014^d^	T	.	0.057	2.3E − 08	0.060	3.4E − 07

^a^Closest gene from variant effect predictor (VEP v98)^60^.

^b^BETA and *P-*value from GWAS on LOAD from Marioni et al.^13^, A1 is the effect allele.

^c^SNPs genome-wide significantly associated with both LOAD AAO and LOAD risk.

^d^SNPs genome-wide significantly associated with LOAD AAO only.

## Discussion

In this study, we investigated the predictive performance of GRS on LOAD using four sets of summary statistics and applied them to three independent datasets. We found a clear pattern in that prediction performance of GRS increases with the use of a more stringent *P-*value threshold for SNP selection and therefore with fewer SNPs in the model. Consistent with simulations and direct estimation (SBayesR), we conclude that a relatively small number (in the hundreds) of common variants contribute to LOAD risk. *APOE* was responsible for most of the prediction accuracy of LOAD, but other variants also show significant prediction accuracy (maximum *R*^2^ on liability scale = 2.0%, 95% bootstrap CI 0.5–4.5%, 1000 replications). Genetic variants that contribute to the risk of disease are also associated with an earlier AAO.

Taking all of our results together, we conclude that the empirical data are consistent with an oligogenic common variant architecture of LOAD (~0.01% of SNPs with MAF > 1% have non-zero effects on LOAD). This is smaller than the polygenicity estimate of 0.26% (s.e. = 0.19%) reported in a previous study^35^. However, considering the standard error of that estimate, it is not significantly (*P* > 0.05) different from our estimate of 0.01% (s.e. = 0.0006%). Besides, this architecture contrasts with many other common diseases and disorders which are highly polygenic. For comparison, we applied the SBayesR method^33^ to GWAS summary statistics for schizophrenia^16,17^, major depression^18^ and Parkinson’s disease^19^, and estimated the proportion of HapMap3 SNPs with non-zero effects size as 17.5% (s.e. = 2.4%), 3.2% (s.e. = 0.8%) and 16.4% (s.e. = 5.8%), respectively. In addition, their optimal *P-*value thresholds of GRS for these diseases were all ≥0.05^16,18,19^. LOAD was previously labelled as polygenic by Escott-Price et al.^20^, who reported a best fitting *P-*value threshold of 0.5. However, most of the control samples (~6000 out of 7277) in their test dataset (Genetic and Environmental Risk in Alzheimer’s Disease consortium) were younger than 60 years old when their disease status was reported, and the ages of most cases were over 75 years^12^. Treating these samples as controls might bias prediction results, since the typical AAO of LOAD is above 65 years. In addition, sample overlap between training and test sets would also lead to a large optimal *P-*value threshold. In Jansen et al.^14^, the best fitting *P-*value threshold was 1.69 × 10^−5^ when the test set was independent of the training set. For a test set that overlaps with the training set (accounting ∼3% of training set^36^), the optimal *P-*value threshold was 0.5. Our simulations show that when the test set is part of a training set, the best *P-*value threshold is close to 1 (Supplementary Fig. 12) (“Methods”), even if the proportion is small (e.g., only 1%), consistent with theory^37^. Therefore, taken together, we conclude that the previous report of LOAD being polygenic is likely biased by sample overlap and/or the ascertainment of controls that may go on to develop LOAD at a later stage.

There is a wide range of LOAD SNP-heritability reported across studies, ranging from 8.9 to 31.2% (Supplementary Table 3). Except for the difference due to the estimation methods, such differences could also be caused by differences in age distributions between datasets (Supplementary Fig. 8), since the genetic effect on LOAD was reported to be age-dependent^38^. Based on the same method, the estimated heritability in datasets with younger individuals was found to be larger than that using older individuals (Supplementary Table 3). Another potential reason could be heterogeneity between datasets, for example with respect to diagnostic criteria. For the summary statistics based on meta-analysis in particular, this heterogeneity would attenuate heritability estimates^5^.

There are a number of limitations in this study: (1) We focused on the additive effect of common variants, and did not explore non-additive genetic or gene by environment effects; (2) our analysis was based on summary statistics from a meta-analysis of a number of datasets. Heterogeneity (e.g., based on different diagnostic criteria) and measurement error (e.g., proxy cases from UKB are self-reported) in these datasets (and those used in this study) might have affected our result. The estimated number of conditionally associated SNPs could be smaller than reported if there is heterogeneity and/or measurement error; (3) the sample sizes of the datasets with real cases and controls used in this study are small, a larger dataset would be required to test the significance of the difference in prediction accuracy (*R*^2^) between GRSs based on optimal *P-*value and other *P-*value thresholds; (4) rare variants were not considered. There are several genes with rare mutations with large effects on LOAD^39–41^. Those mutations contribute little to heritability and to prediction accuracy in population samples because of their low frequency. Larger GWAS samples should allow identification of the remaining undiscovered common SNPs associated with LOAD but also offer the opportunity to identify rarer SNPs (e.g., MAF in 0.001–0.1) in order to refine and improve the GRS.

## Methods

### Study populations

*AIBL*: we selected 216 cases and 631 controls (participants with mild cognitive impairment were regarded as controls) with genotype information from the Australian Imaging, Biomarker & Lifestyle Flagship Study (Table 1). We removed SNPs with minor allele frequency smaller than 0.01, SNP missingness rate larger than 0.05, and not passing Hardy–Weinberg equilibrium test (*P* < 5 × 10^−6^). Genotypes were imputed to the sequencing data from the Haplotype Reference Consortium (r1.1) using the Sanger Imputation Service (https://imputation.sanger.ac.uk). A total of 6,972,431 SNPs with info score larger than 0.8 were selected after imputation. Data were collected by the AIBL study group. AIBL study methodology and acquisition of genetic data have been reported previously^42,43^. Ethics approval for the AIBL study and all experimental protocols were provided by the ethics committees of Austin Health, St Vincent’s Health, Hollywood Private Hospital and Edith Cowan University. Informed consent was obtained from all participants.

*Sydney MAS*: we selected 77 cases and 588 controls (including participants with mild cognitive impairment) with genotype information from the Sydney Memory and Ageing Study^44^ (Table 1). We applied the same quality control steps and imputation as in that in AIBL. In total, 4,303,719 SNPs with info score larger than 0.8 were selected after imputation. Acquisition of genetic data has been described previously^45^. Informed consent was obtained from all participants, and Sydney MAS was approved by the Human Research Ethics Committee of the University of New South Wales (# HC14327).

*UKB family history*: UKB data (http://www.ukbiobank.ac.uk) were collected on over 500,000 individuals aged between 37 and 73 years from across Great Britain (England, Wales and Scotland) at the study baseline (2006–2010), including health, cognitive and genetic data. Family history of AD was ascertained via self-report. Participants were asked “Has/did your father ever suffer from Alzheimer’s disease/dementia?” (Data-Field: 20107) and “Has/did your mother ever suffer from Alzheimer’s disease/dementia?” (Data-Field: 20110). Self-report data from the initial assessment visit (2006–2010), the first repeat assessment visit (2012–2013) and the imaging visit (2014+) were aggregated. We only included participants with parents older than 60 years or whose parents died after 60 years of age. Only genetically unrelated individuals (genetic relationship correlation <0.05) with European ancestry were selected. In total, 22,557/13,118 individuals with maternal/paternal LOAD were selected as proxy case samples, 231,767/241,206 individuals without maternal/paternal LOAD were selected as proxy control samples. Imputation and QC steps on SNPs have been detailed elsewhere^46^, 8,545,378 SNPs left after QC.

*UKB*: additional information on LOAD was obtained for participants themselves from UKB. Briefly, 383 participants with a diagnosis of “Alzheimer’s disease” (ICD10 code: G30.1 and G30.9) or “Dementia in Alzheimer’s disease” (ICD10 code: F00.1 and F00.9) or “dementia/Alzheimer’s/cognitive impairment” (UKB Data-Coding 6: 1263) were selected. We randomly selected 1915 participants (with age at baseline greater than 60) from the remaining samples as controls. These samples were used as a test set. Informed consent was obtained by UKB from all participants, and the ethics approval for the UKB study was obtained from the North West Centre for Research Ethics Committee (11/NW/0382).

### The estimation of intercept for LDSC

An inaccurately estimated intercept in LDSC could affect the precision of the estimate of the genetic correlation^29^. We therefore calculated the intercept directly other than estimating it in LDSC. The intercept was calculated as $\frac{{N_s}}{{\sqrt {N_1N_2} }}$, *N*_1_ and *N*_2_ are the average per SNP sample size in each study, *N*_*s*_ is the number of overlapping samples between studies. The intercept between Marioni et al. (UKB)^13^ and Marioni et al. (meta)^13^ was estimated to be 0.75 (it was 0.77 from LDSC), and the intercept between Lambert et al. (stage 1)^12^ and Marioni et al. (meta)^13^ was 0.67 (it was 0.68 from LDSC).

### Heritability and prediction accuracy on liability scale

The heritability on liability scale ($h_{\rm{SNP(l)}}^2$) can be transformed from heritability on observed scale ($h_o^2$, treating case/control as 1/0)^47^:

1 $$h_l^2 = h_o^2\frac{{K(1 - K)}}{{z^2}}\frac{{K(1 - K)}}{{P(1 - P)}},$$

where *K* is the population disease prevalence, *P* is the proportion of cases in the ascertained sample and *z* is the height of the standard normal probability density function at the truncation threshold *t* which corresponds to probability *K*. *z* can be calculated using the R functions *qnorm()* and *dnorm()*: *t* *=* *qnorm(1* *−* *K)* and *z* *=* *dnorm(t)*. The formula is more complicated for transforming prediction accuracy on the observed scale ($R_o^2$) to the liability scale ($R_l^2$)^48^:

2 $$R_l^2 = \frac{{R_o^2C}}{{1 + \theta R_o^2C}},$$

where *C* is $\frac{{K(1 - K)}}{{z^2}}\frac{{K(1 - K)}}{{P(1 - P)}}$ and *θ* is $\frac{{z\left( {P - K} \right)}}{{K\left( {1 - K} \right)}}(\frac{{z\left( {P - K} \right)}}{{K\left( {1 - K} \right)}} - t)$. We used 5% as the population disease lifetime prevalence in this study^49^.

The following equation was used to transform $h_{{\rm{SNP}}(l_{K1})}^2$ estimated using population prevalence *K*1 to $h_{{\rm{SNP}}(l_{K2})}^2$ using population prevalence *K*2:

3 $$h_{{\rm{SNP}}(l_{K2})}^2 = h_{{\rm{SNP}}(l_{K1})}^2 \times \left( {\frac{{z_{K1}}}{{z_{K2}}}\frac{{K2(1 - K2)}}{{K1(1 - K1)}}} \right)^2,$$

where $z_{K1}$ and $z_{K2}$ are the values of the standard normal probability density function at the truncation threshold *z*-score, which corresponds to probabilities *K*1 and *K*2.

### Genetic correlation

The genetic correlation between two sets of summary statistics was estimated using LDSC^50^. To avoid the potential effect of *APOE* in determining the genetic correlation, we used the flag “--two-step 30” to remove SNPs with a chi-square test statistic larger than 30 (corresponds to a genome-wide significant *P-*value of 5 × 10^−8^) in either study. Note that this is the default option for univariate LDSC analyses.

### Simulation of a trait with different number causal SNPs (one of the SNPs is a major mutation)

We randomly selected 100,000 unrelated individuals from UKB. We simulated a trait with heritability 0.2 using different number of causal SNPs (2^4^,2^5^,2^6^,2^7^,2^8^,2^9^,2^10^,2^11^,2^12^,2^13^,2^14^) randomly selected from 1,056,156 SNPs. We chose one of the selected SNPs as a major mutation, and assumed that it explained 20, 50 and 80% of the heritability. For each simulated trait with a certain number of causal SNPs, we selected 10,000 individuals as a test set and chose 10,000–90,000 individuals from the remaining individuals as a training set. We performed a GWAS on the training set and examined the prediction performance of GRS on the test set. GRS were calculated based on near-independent SNPs selected from 80 different *P-*value thresholds (from 1 × 10^−8^ to 1) and LD clumping (*R*^2^ = 0.01, region = 1 Mbp). The optimal value was selected as the *P-*value threshold that maximised the prediction accuracy.

### Simulation of a trait with different number causal SNPs (no major mutation)

We randomly selected 100,000 unrelated individuals from UKB. We simulated a trait with heritability 0.06 using different number of causal SNPs (2^4^,2^5^,2^6^,2^7^,2^8^,2^9^,2^10^,2^11^,2^12^,2^13^,2^14^,2^15^,2^16^,2^17^) randomly selected from 1,037,804 SNPs. For each simulated trait with a certain number of causal SNPs, we selected 10,000 individuals as a test set and chose 10,000–90,000 individuals from the remaining individuals as a training set. We performed a GWAS on the training set and examined the prediction performance of the GRS on the test set. GRS were calculated based on near-independent SNPs selected from 80 different *P-*value thresholds (from 1 × 10^−^^8^ to 1) and LD clumping (*R*^2^ = 0.01, region = 1 Mbp).

### Estimating the number of SNPs with non-zero effect on LOAD

We used SBayesR^33^ (implemented in GCTB^51^) to estimate the number of SNPs with a non-zero effect on LOAD. We used the GWAS summary statistics based on the meta-analysis from Marioni et al.^13^ (the sum of the number of participants in IGAP1 and IGAP2 and 25% of the number of maternal and paternal samples was used as the sample size). Summary statistics from Jansen et al.^14^ was not utilised since the weights used to generate these summary statistics (in the meta-analysis) were not optimal. The model did not converge while using summary statistics from Lambert et al.^12^. The estimated number of SNPs (excluding SNPs from chromosome 19) with non-zero effect based on summary statistics from Marioni et al. (UKB)^13^ was 325 (s.e. = 69). The number was larger than that from Marioni et al. (meta)^13^ since the disease status in UKB was reported but not diagnosed. Therefore, SNPs associated with other diseases might also be detected. The LD matrix was calculated based on 1,056,156 SNPs (1,056,154 HapMap3 SNPs and two *APOE* SNPs: rs429358 and rs7412) using a random sample of 10,000 unrelated (genetic relatedness <0.05) individuals in the UKB. We set the starting values (*π*) for each mixture component to 0.95, 0.03, 0.01 and 0.01, respectively, and their corresponding gamma values to 0, 0.01, 0.1 and 1. *π* are probabilities of the SNP in the mixture classes and the gamma coefficients constrain how the common marker effect variance scale in each class. The total number of iterations for the MCMC chain was set to 50,000. We used the same parameters for the GWAS summary statistics of the other disorders considered: Parkinson’s disease^52^, major depression^53^ and schizophrenia^17^. In addition, we removed SNPs from chromosome 19 and performed the analysis with the same parameters on the remaining 1,037,804 SNPs.

### Genetic risk score based on LDpred

We randomly selected 10,000 unrelated (genetic relatedness <0.05) individuals from UKB as the LD reference of 1,037,804 SNPs (all HapMap3 SNPs excluding SNPs from chromosome 19). We examined the prediction accuracy of GRSs by assigning 14 proportions of causal SNPs: 1 × 10^−8^, 1 × 10^−7^, 1 × 10^−6^, 1 × 10^−^^5^, 3 × 10^−5^, 1 × 10^−4^, 3 × 10^−4^, 1 × 10^−3^, 3 × 10^−3^, 0.01, 0.03, 0.1, 0.3, 1.

### Genome-wide survival analysis on AAO of LOAD

Two types of parental age were used in the GWSA as parental proxy AAO of LOAD: parental age at death and parental age at measurement. We performed GWSA on maternal and paternal AAO of LOAD separately. Specifically, we used Cox proportional hazard models^24^ implemented in the “survival” R package^54^ to identify SNPs associated with parental AAO of LOAD across the genome. Compared to normal GWSA that detect the SNP effect on AAO of individuals themselves, we expect the effect size from GWSA on parental AAO to be halved^25^. The Cox model is defined as:

4 $$h\left( t \right) = h_0(t)\exp (\beta _0SNP + {\boldsymbol{\beta}}\ {\boldsymbol{COV}}),$$

where $h\left( t \right)$ is the hazard rate of developing LOAD at age *t*, *t* is the proxy parental AAO for cases and parental age at last assessment for controls. *h*_0_(*t*) is the baseline hazard of developing LOAD, which is not estimated in Cox regression. *β*_*0*_ is the effect of a SNP on the hazard ratio (HR) and ***β*** are effects of covariates (***COV***), including assessment centre, genotype chip array, age of participants, 20 genetic principal components (PCs), and whether the parent is alive or not.

Based on GWSA results on maternal AAO and paternal AAO, we carried out an inverse-variance meta-analysis using METAL^55^ and identified six independent (pairwise LD < 0.01) genome-wide significant (*P* < 5 × 10^−8^) loci (Supplementary Fig. 11a).

The effect size log(HR) and standard error of each SNP in our survival analysis on parental AAO of LOAD were multiplied by 2, so that it can be on the same scale as a traditional design (i.e., survival analysis on AAO of LOAD using individual-level data)^13,25,56^. After meta-analysis with these summary statistics, we identified SNPs in 16 loci that were genome-wide significantly (*P* < 5 × 10^−8^) associated with LOAD AAO (Table 2 and Supplementary Fig. 11b).

The Cox model assumes proportional hazards. We examined whether the assumption was violated in the 16 genome-wide SNPs by investigating the association between Schoenfeld residuals from the model and age^57^. The significant association suggests a non-constant HR. The SNP effects on both maternal and paternal AAO of AD were tested. We used the cox.zph function in the R “survival” package^54^ to calculate the significance of this association. rs1081105 (*APOE*) based on maternal AD AAO was found to be significant (*P* < 0.05/32), suggesting the HR of this SNP is not constant with time (Supplementary Fig. 13), there is SNP by age effect. Given that the HR of this SNP was extremely large (HR = 2.6) and significant (*P* = 4.0 × 10^−106^, Cox proportional hazards model), we retained this SNP in the model.

### Effect of major mutation on the estimation of SNP-based heritability

We randomly selected 40,000 unrelated individuals from the UKB. We simulated a trait with heritability 0.2 and 100 causal SNPs randomly selected from 1,056,156 SNPs (1,056,154 HapMap3 SNPs and two *APOE* SNPs: rs429358 and rs7412). One of the randomly chosen SNPs was set to be a major mutation. The proportion of heritability explained by this SNP varied from 0 to 100%. For each proportion (e.g., 50%), we iterated the following steps 100 times: (1) select 100 SNPs and choose one as the major SNP; (2) generate a continuous trait with heritability 0.2 using the standardised dosage of the 100 SNPs (with effect sizes of 99 SNPs sampled from a standard normal distribution and the effect size of the major variant calculated to make sure it explained a specific proportion (e.g., 50%) of SNP-based heritability); (3) perform GWAS on the simulated trait with 20 genetic PCs as covariates; (4) use LDSC to estimate the heritability based on the GWAS summary statistics. Both default setting (SNPs with *χ*^2^ > 30 are removed) and using all SNPs (SNPs with *χ*^2^ > 20,000 are removed) were examined; (5) use GCTA–GREML to estimate the heritability based on the individual-level data with 20 PCs as covariates.

### Estimating per SNP sample size

In logistic regression, the sample size of each SNP (*x*) can be estimated based on the standard error (s.e.) of log(odds ratio)^58^:

5 $$N \approx \frac{{{\mathrm{var}}(y)}}{{{\rm{s.e.}}^2{\mathrm{var}}(x)}} \approx \frac{1}{{2Np(1 - p)P(1 - P)}},$$

where *P* is the proportion of cases, *p* is the minor allele frequency and *y* is the disease (1 for case and 0 for control). We define *P* as 0.5 so that it is the sample size for a balanced design.

### Relationship between sample overlap and prediction pattern

We randomly selected 90,000 unrelated individuals from UKB to simulate a trait with heritability 0.2 and 128 causal SNPs (close to the estimated number SNPs with non-zero effect on LOAD) selected from 1,056,156 SNPs (1,056,154 HapMap3 SNPs and two *APOE* SNPs: rs429358 and rs7412). We chose one of the selected SNPs as a major mutation, and assumed that it explained 20, 50 and 80% of the heritability. We performed GWAS on these individuals (training dataset) to get the summary statistics. We randomly selected a proportion of individuals from the training dataset (fraction ranges from 1 to 20%) as a test set and examined the prediction pattern of GRS (based on the GWAS summary statistics) on this test set.

### Reporting summary

Further information on research design is available in the Nature Research Reporting Summary linked to this article.

## Supplementary information

Supplementary Information

Peer Review File

Reporting Summary

## Data Availability

The genotype data used in this work were obtained from the UK Biobank, the Australian Imaging, Biomarker & Lifestyle Flagship Study and the Sydney Memory and Ageing Study. GWAS summary statistics on late-onset Alzheimer’s disease (LOAD) are available at Marioni et al.^13^ [https://cnsgenomics.com/content/data], Lambert et al.^12^ [http://web.pasteur-lille.fr/en/recherche/u744/igap/igap_download.php] and Jansen et al.^14^ [https://ctg.cncr.nl/software/summary_statistics]. Survival GWAS summary statistics on age at onset of LOAD are available at Huang et al.^28^ [https://www.niagads.org/datasets/ng00058]. Summary statistics from the meta-analysis on LOAD AAO are available at [https://cnsgenomics.com/content/data]. The data that support the findings of this study are available from UK Biobank (http://www.ukbiobank.ac.uk/about-biobank-uk/). Restrictions apply to the availability of these data, which were used under license for the current study (Project ID: 12505). Data are available for bona fide researchers upon application to the UK Biobank. All other data are contained in the article and its Supplementary information, or are available on request.
