# Supplementary material for: Risk prediction of late-onset Alzheimer’s disease implies an oligogenic architecture
Source: Nat Commun. 2020 Sep 23;11:4799. doi: 10.1038/s41467-020-18534-1 (PMC7511365; doi:10.1038/s41467-020-18534-1)
Supplement: Supplementary file 1 — Supplementary Information [file 41467_2020_18534_MOESM1_ESM.docx]

**Supplementary Information**

**Risk prediction of late-onset Alzheimer’s disease implies an oligogenic architecture**

Zhang et al.

**Supplementary Table 1.** The genetic correlations between four summary statistics

|  | **Lambert et al. (stage 1) ^1^** | **Marioni et al. (meta) ^2^** | **Jansen et al. (meta) ^3^** |
| --- | --- | --- | --- |
| **Marioni et al. (UKB) ^2^** | 0.92 (0.15) | 0.76 (0.05) | 0.99 (0.04) |
| **Lambert et al. (stage 1) ^1^** |  | 1.27 (0.06) | 0.95 (0.07) |
| **Marioni et al. (meta) ^2^** |  |  | 0.93 (0.02) |

**Supplementary Table 2.** 22 SNPs used to generate the GRS_full_ with largest prediction accuracy.

| **CHR** | **BP** | **SNP** | **A1** | **Closest Gene ^(1)^** | **Beta ^(2)^** | **P-value** |
| --- | --- | --- | --- | --- | --- | --- |
| 1 | 207692049 | rs6656401 | A | CR1 | 0.14 | 1.4E-29 |
| 2 | 127894615 | rs744373 | A | BIN1 | -0.14 | 5.2E-40 |
| 2 | 234003359 | rs7419666 | T | INPP5D | -0.06 | 4.1E-10 |
| 6 | 47432637 | rs9381563 | T | . | -0.08 | 5.8E-14 |
| 7 | 100004446 | rs1476679 | T | ZCWPW1 | 0.09 | 9.9E-19 |
| 7 | 143099107 | rs7791765 | T | EPHA1 | 0.09 | 7.1E-14 |
| 8 | 27220310 | rs17057043 | A | PTK2B | 0.08 | 3.0E-16 |
| 8 | 27464519 | rs11136000 | T | CLU | -0.11 | 4.2E-28 |
| 10 | 11720308 | rs7920721 | A | AL512631.1 | -0.07 | 3.2E-11 |
| 11 | 47449072 | rs12292911 | A | PSMC3 | 0.06 | 3.3E-09 |
| 11 | 59942815 | rs7935829 | A | MS4A6A | 0.09 | 2.0E-18 |
| 11 | 85868640 | rs3851179 | T | RNU6-560P | -0.12 | 4.3E-35 |
| 11 | 121435587 | rs11218343 | T | SORL1 | 0.21 | 4.6E-17 |
| 14 | 53400629 | rs17125944 | T | FERMT2 | -0.11 | 4.1E-11 |
| 14 | 92931737 | rs941648 | A | SLC24A4 | -0.07 | 8.6E-11 |
| 15 | 59045774 | rs593742 | A | ADAM10 | 0.07 | 2.8E-11 |
| 17 | 5137047 | rs7225151 | A | SCIMP | 0.10 | 6.1E-12 |
| 17 | 61536308 | rs9896864 | A | AC005828.5 | -0.21 | 5.6E-09 |
| 19 | 1039444 | rs3795065 | T | CNN2 | -0.08 | 3.7E-12 |
| 19 | 45412079 | rs7412 | T | APOE | -0.44 | 4.0E-82 |
| 19 | 45411941 | rs429358 | T | APOE | -1.13 | 0 |
| 20 | 54984768 | rs6064392 | T | CASS4 | -0.11 | 6.4E-10 |

^(1)^ Closest gene is from Variant Effect Predictor (VEP v98) ^4^

^(2)^ Beta is from the meta-analysis from Marioni et al. ^2^

**Supplementary Table 3.** SNP heritability reported in different studies and their transformed value by assuming a disease population prevalence of 5%.

| **Source** | **Data set** | **N_case_/N_cont_** | **Mean Age** | **prevalence ^(1)^** | $\boldsymbol{h}_{\boldsymbol{l}}^{\boldsymbol{2}}$ **(s.e.) ^(1)^** | **Current prevalence** | **Transformed** $\boldsymbol{h}_{\boldsymbol{l}}^{\boldsymbol{2}}$ **(s.e.)** | **Method** |
| --- | --- | --- | --- | --- | --- | --- | --- | --- |
| Lee et al. ^5^ | GERAD ^(2)^ | 3,290/3,849 | ~64.3 ^(5)^ | 0.02 | 24% (3%) | 0.05 | 31% (4%) | GCTA-GREML ^6^ |
| Ridge et al. ^7^ | ADGC ^(3)^ | 5,708/5,214 | 75.1 | 0.13 | 33% (3%) | 0.05 | 25% (2%) | GCTA-GREML |
| Brainstorm et al. ^8^ | IGAP ^(4)^ | 17,008/37,154 | ~71.2 ^(6)^ | 0.17 | 13% (3%) | 0.05 | 9% (2%) | LDSC ^9^ |
| Brainstorm et al. ^8^ | GERAD | 3,941/7,848 | 63.3 ^(7)^ | 0.17 | 25% (4%) | 0.05 | 17% (3%) | LDSC |

1. $h_{l}^{2}$ and population disease prevalence reported in original study.
2. GERAD: Genetic and Environmental Risk in Alzheimer’s Disease (GERAD) consortium.
3. ADGC: Alzheimer’s Disease Genetics Consortium.
4. IGAP: International Genomics of Alzheimer's Project (stage 1)
5. The mean age of samples is approximated as the weighted mean age collected from Supplementary Table 1 of Harold et al. ^10^.
6. The mean age of samples is approximated as the weighted mean age collected from Supplementary Table 1 of Jansen et al. ^3^. Age at onset of case was regarded as a proxy of age at measurement.
7. The mean age of samples is calculated as the weighted mean age collected from Supplementary Table 1 of Harold et al. ^10^.


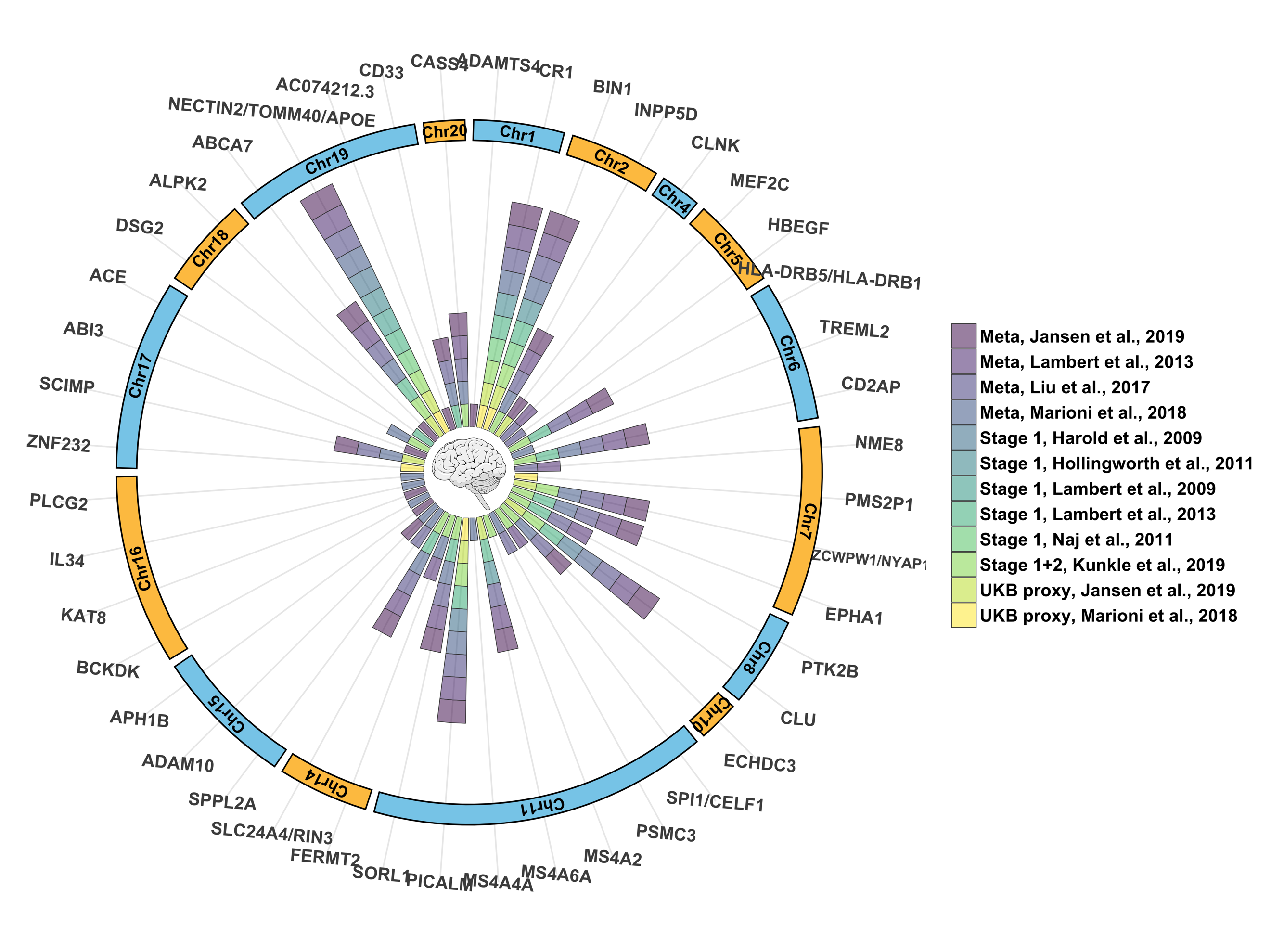


**Supplementary Figure 1**: The genes are collected from different studies. They are the closest genes to SNPs (minor allele frequency > 0.01) genome-wide significantly (P < 5x10^-8^) associated with LOAD in each study ^1-3,10-14^. It should be noted that the discovery samples between these studies are not independent.


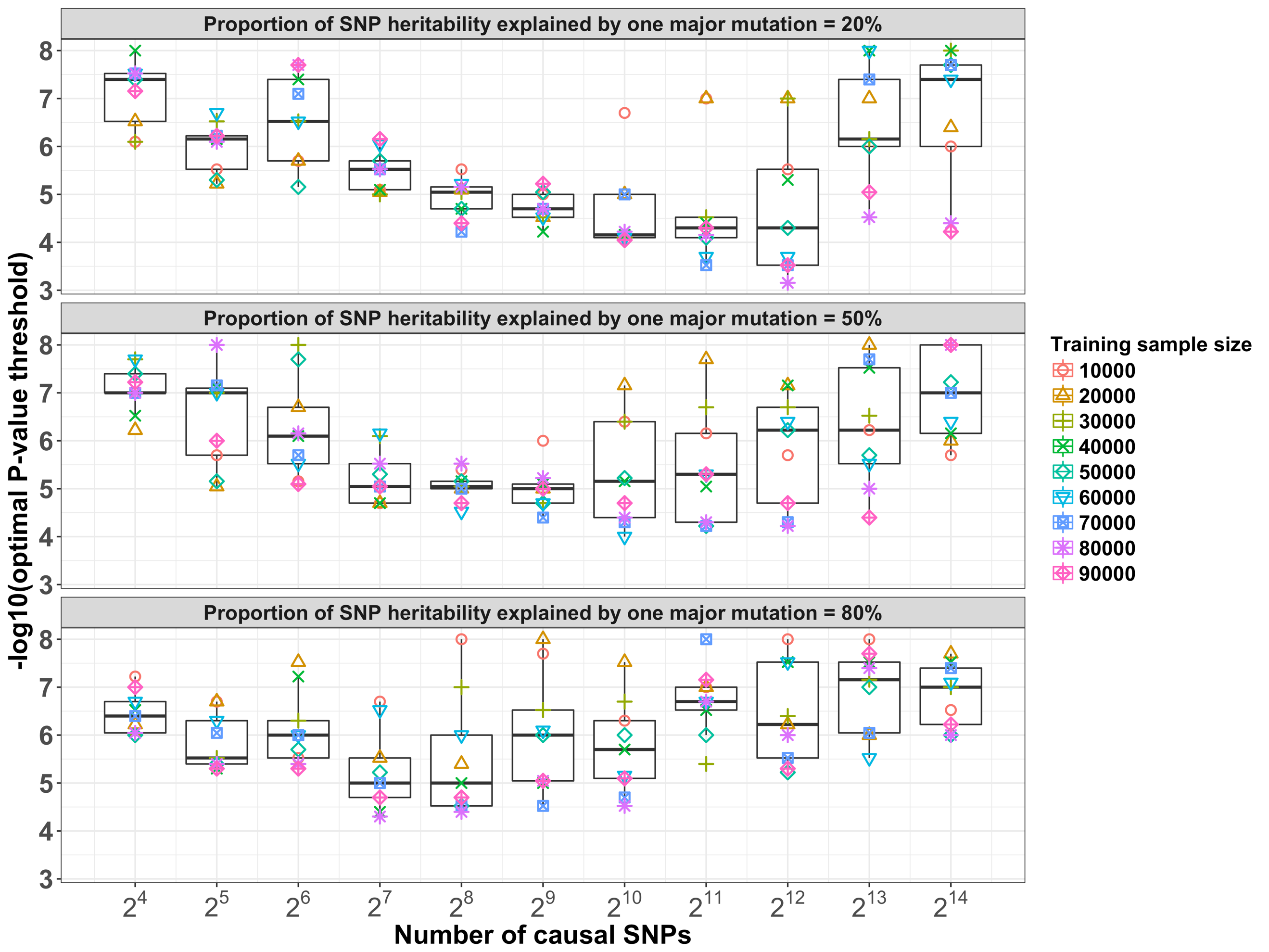


**Supplementary Figure 2**: The relationship between optimal P-value threshold of genetic risk score and number of causal SNPs (M_causal_). For each scenario, we generated a phenotype of 100,000 individuals based on a specified M_causal_ (e.g., 128) with heritability 0.2. One of the causal SNPs was set to explain 20%, 50% or 80% of the heritability. We randomly selected 10,000 individuals as the test set. Based on the unselected individuals, we randomly chose 10,000, 20,000, 30,000, 40,000, 50,000, 60,000, 70,000, 80,000 and 90,000 individuals separately as training sets and used them to perform GWAS. We examined the performance of genetic risk score (based on LD clumping with 80 separate P-value thresholds) on the test set (N_test_ = 10,000), and selected the optimal P-value threshold. Box plot shows the median (centre line), the interquartile range (box), and whiskers (±1.5 times interquartile range).


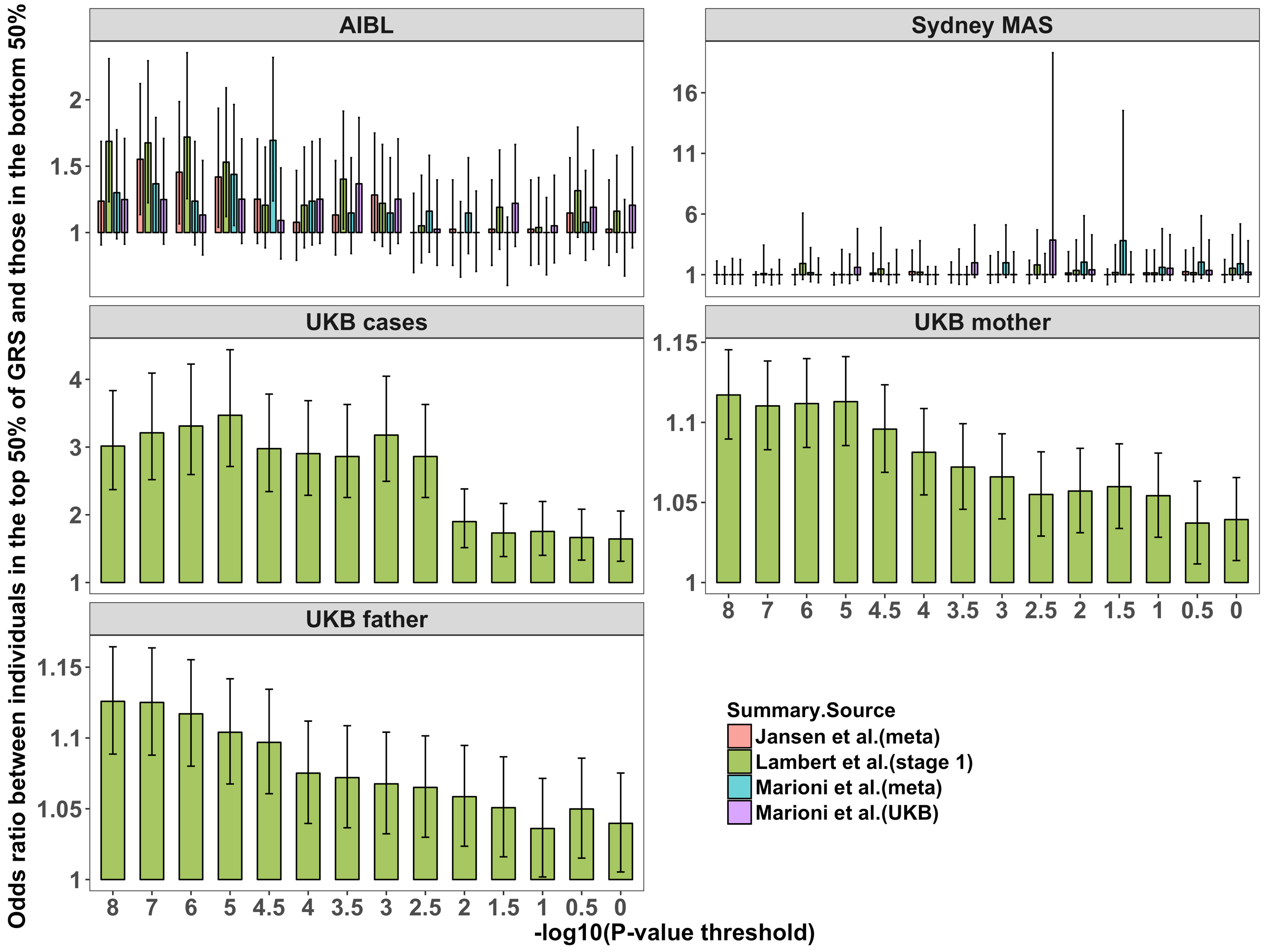


**Supplementary Figure 3**: The odds ratio of individuals in the top 50% of GRS_no19_ (based on SNPs selected using different P-value thresholds) and those in the bottom 50%. GRS_no19_ was calculated based on all HapMap3 SNPs but excluding SNPs from chromosome 19. GRS_no19_ on samples from UKB was based on summary statistics from Lambert et al. (stage 1) ^1^. The error bars represent 95% confidence interval.


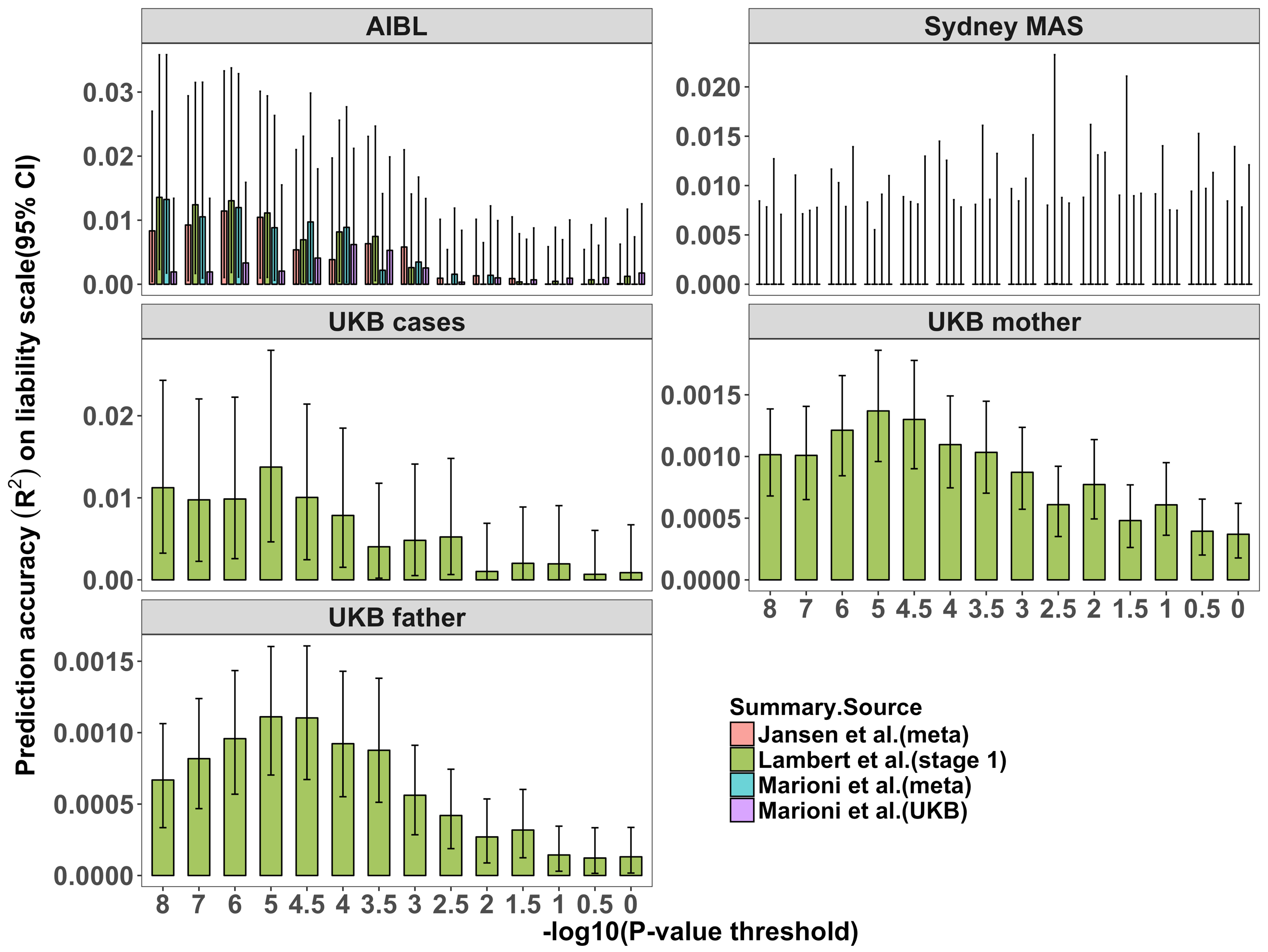


**Supplementary Figure 4**: The prediction accuracy of GRS based on SNPs selected using different P-value thresholds in LD-clumping (R^2^ = 0.2, window size = 1Mbp). GRS_no19_ is calculated based on HapMap3 SNPs but excluding SNPs from chromosome 19. Prediction results on samples from UKB and UKB parents are based on summary statistics from Lambert et al. (stage 1)^1^ only. The error bars represent 95% confidence interval and the confidence interval of each R^2^ was calculated based on bootstrap with 1,000 replications^15^.


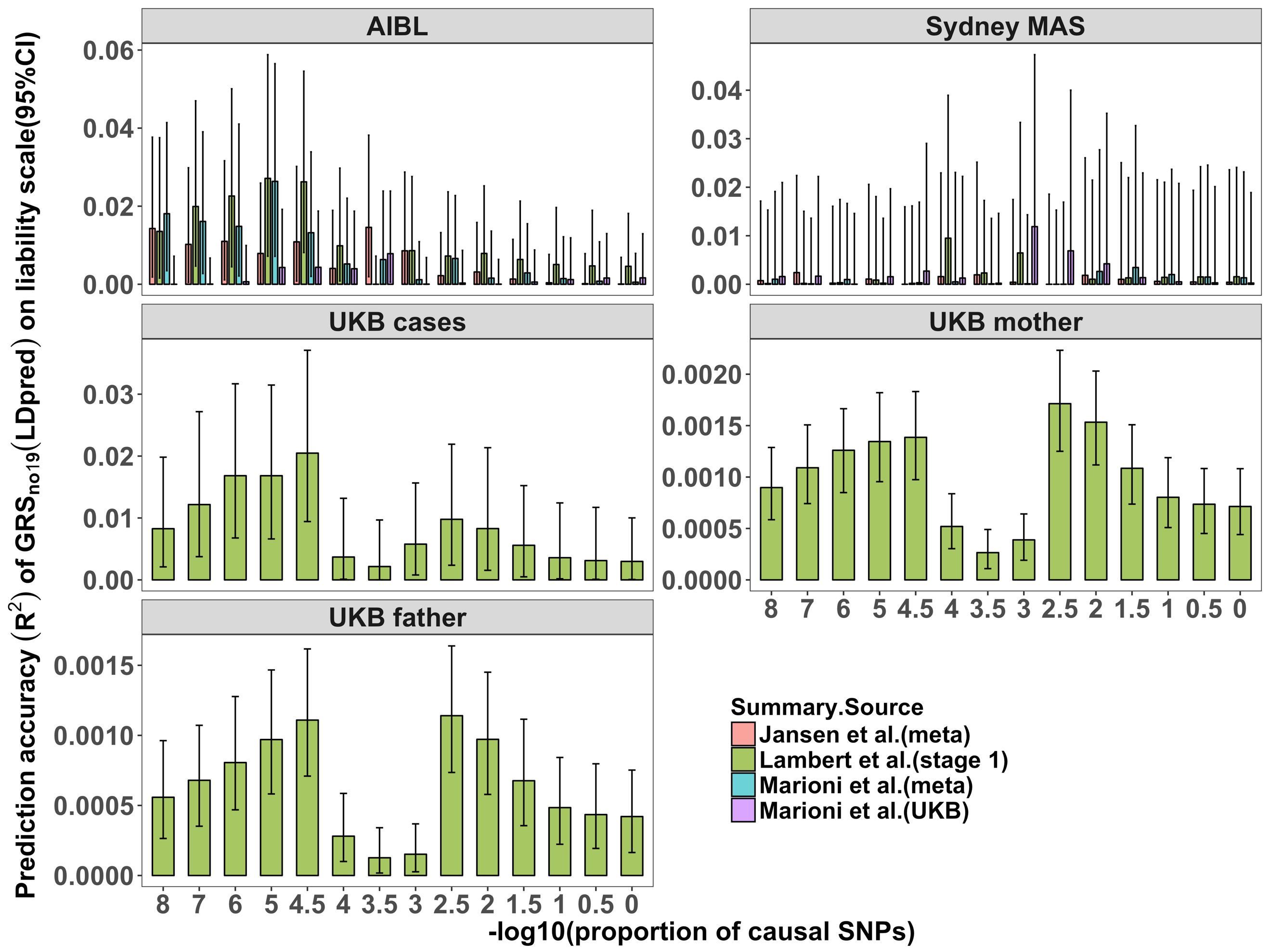


**Supplementary Figure 5**: The prediction accuracy of GRS_no19_ using all HapMap3 SNPs but excluding SNPs from chromosome 19 with effect sizes re-estimated based on LDpred. Prediction results on samples from AIBL and Sydney MAS were based on four sets of summary statistics. Prediction result on samples from UKB was based on summary statistics from Lambert et al. (stage 1) ^1^. Different proportions of causal SNPs were set in LDpred to compare the prediction performance. The error bars represent 95% confidence interval , and the confidence interval of each R^2^ was calculated based on bootstrap with 1,000 replications ^15^.

**
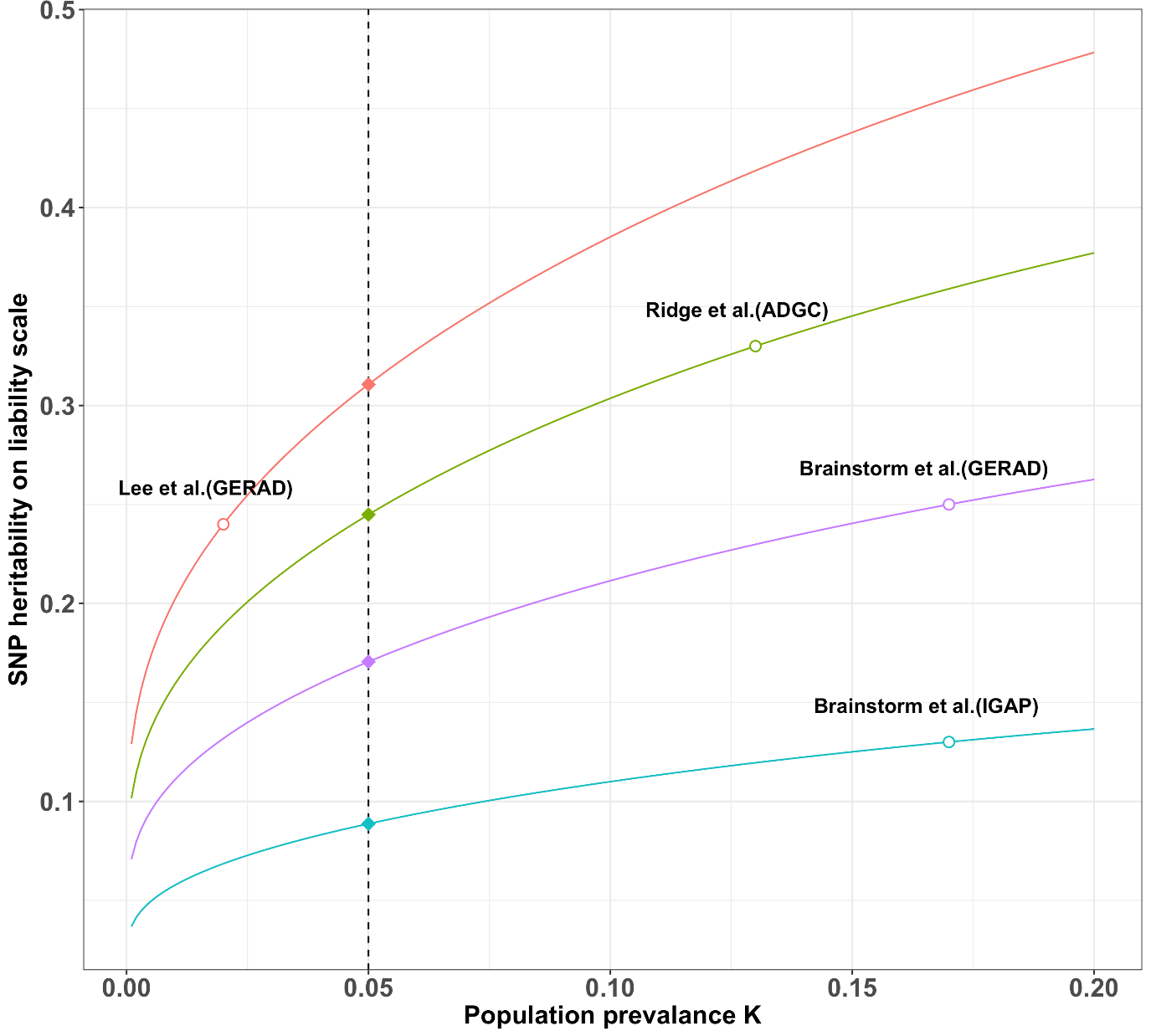
**

**Supplementary Figure 6**: SNP heritability reported in different studies and their transformed value by assuming different disease population prevalence. The dashed vertical line represents disease population prevalence 0.05.


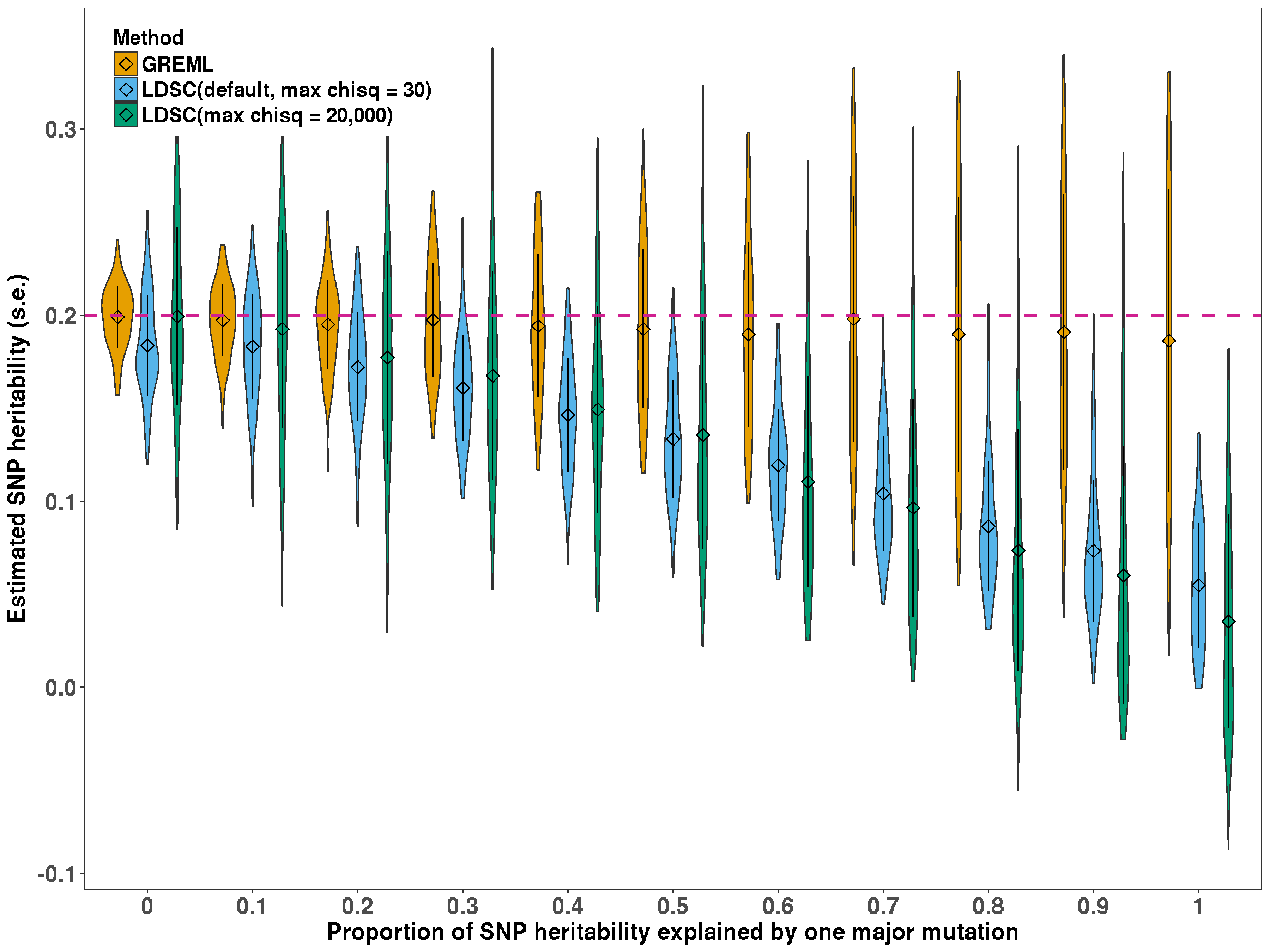


**Supplementary Figure 7**: The effect of major mutation on the estimation of SNP heritability by different methods. The red dash line represents the simulated heritability 0.2. x-axis represents the proportion of heritability explained by the major mutation, y-axis is the estimated SNP heritability by different methods. There are 100 replicates in each scenario. The diamond is the average estimated value in each scenario. Standard error of the average value is calculated as the standard deviation of 100 estimates in each scenario.

**
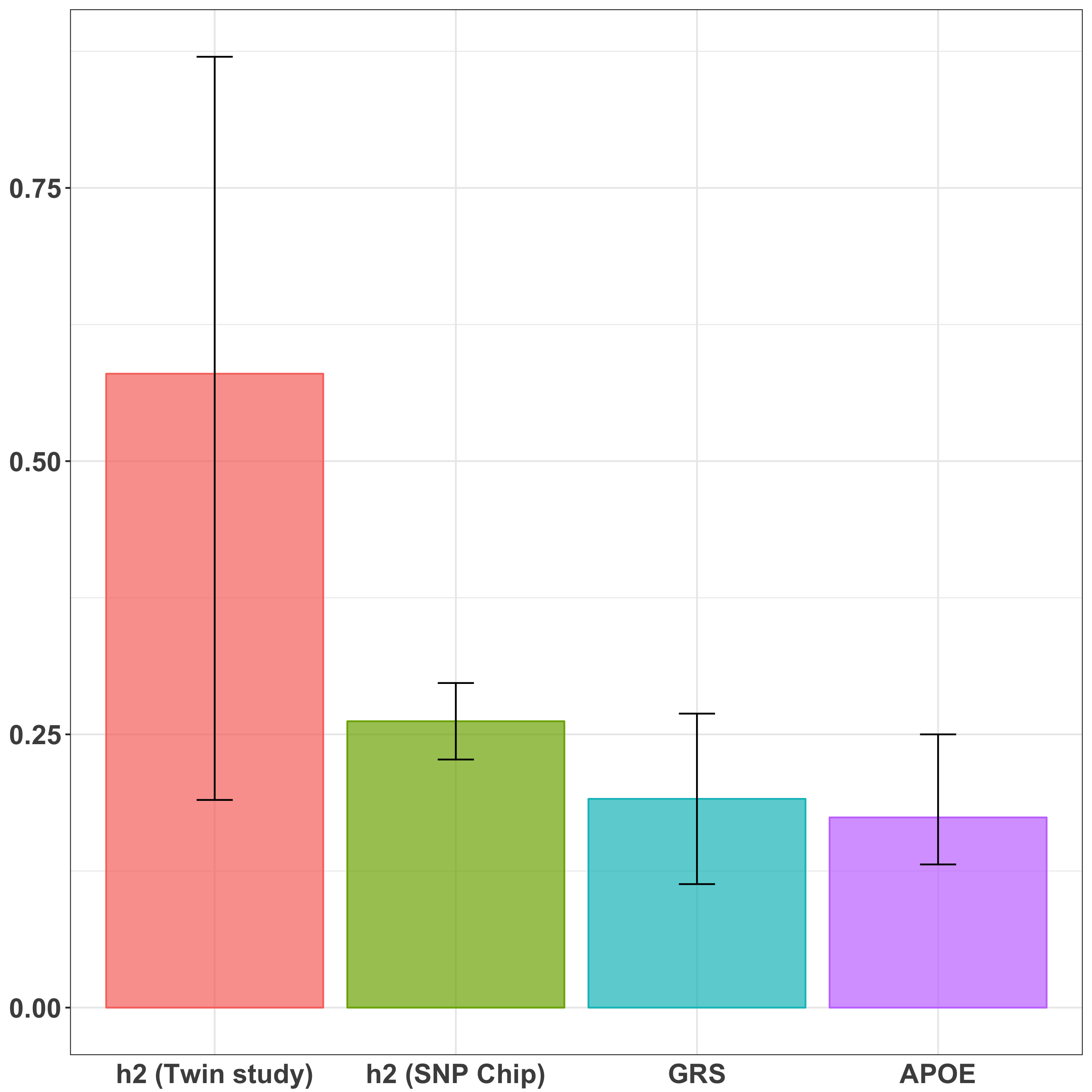
**

**Supplementary Figure 8**: The heritability estimated based on twin study/SNP chip and prediction accuracy of GRS (based on common SNPs) and *APOE*. The error bars represent 95% confidence interval.


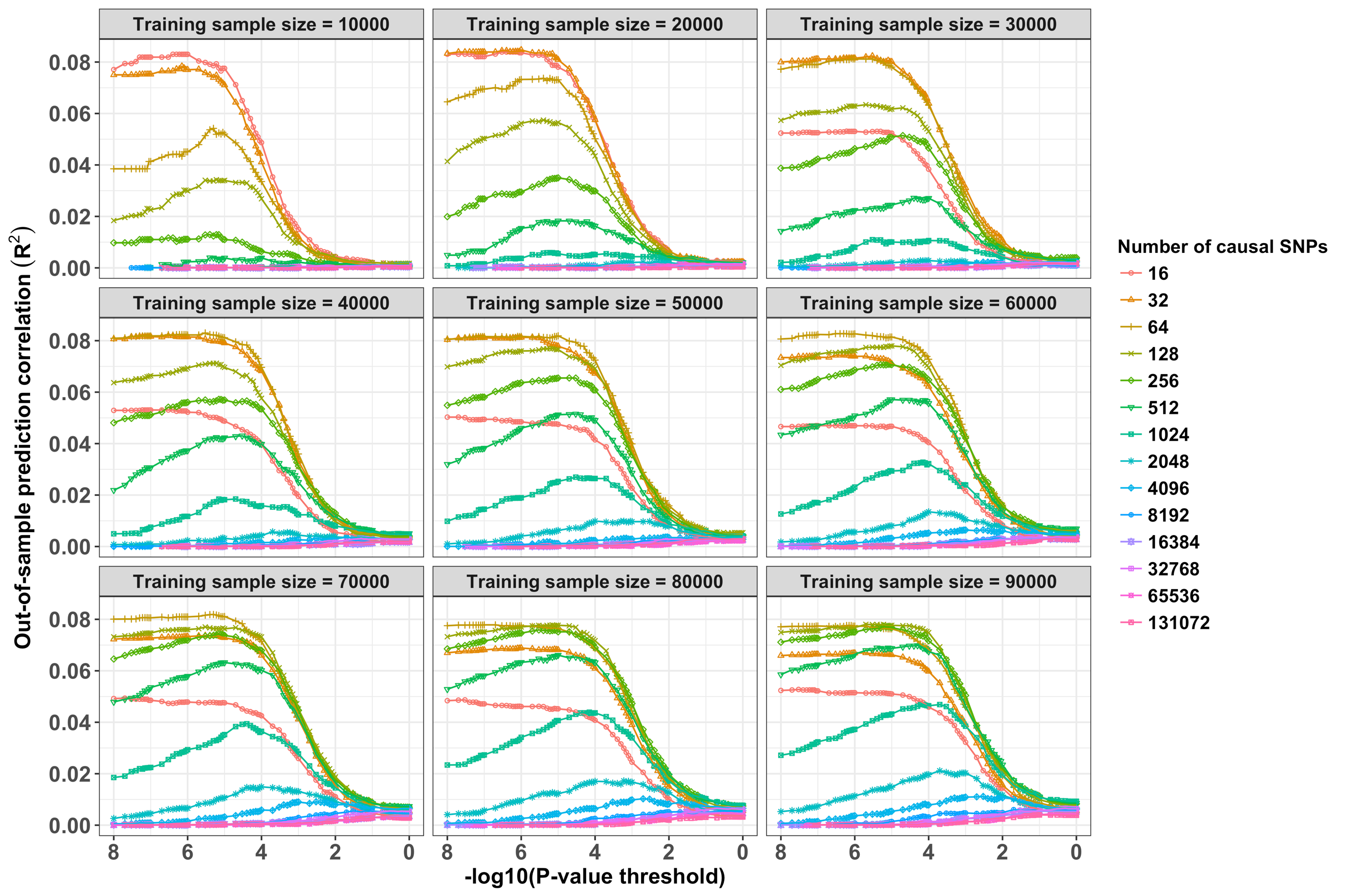


**Supplementary Figure 9**: The relationship between optimal P-value threshold of genetic risk score and number of causal SNPs (M_causal_). For each scenario, we generated a phenotype of 100,000 individuals based on a specified M_causal_ (e.g., 128) with heritability 0.09. We randomly selected 10,000 individuals as the test set. Based on the unselected individuals, we randomly chose 10,000, 20,000, 30,000, 40,000, 50,000, 60,000, 70,000, 80,000 and 90,000 individuals separately as training sets and used them to perform GWAS. We examined the performance of genetic risk score (based on the GWAS summary) on the test set (N_test_ = 10,000).


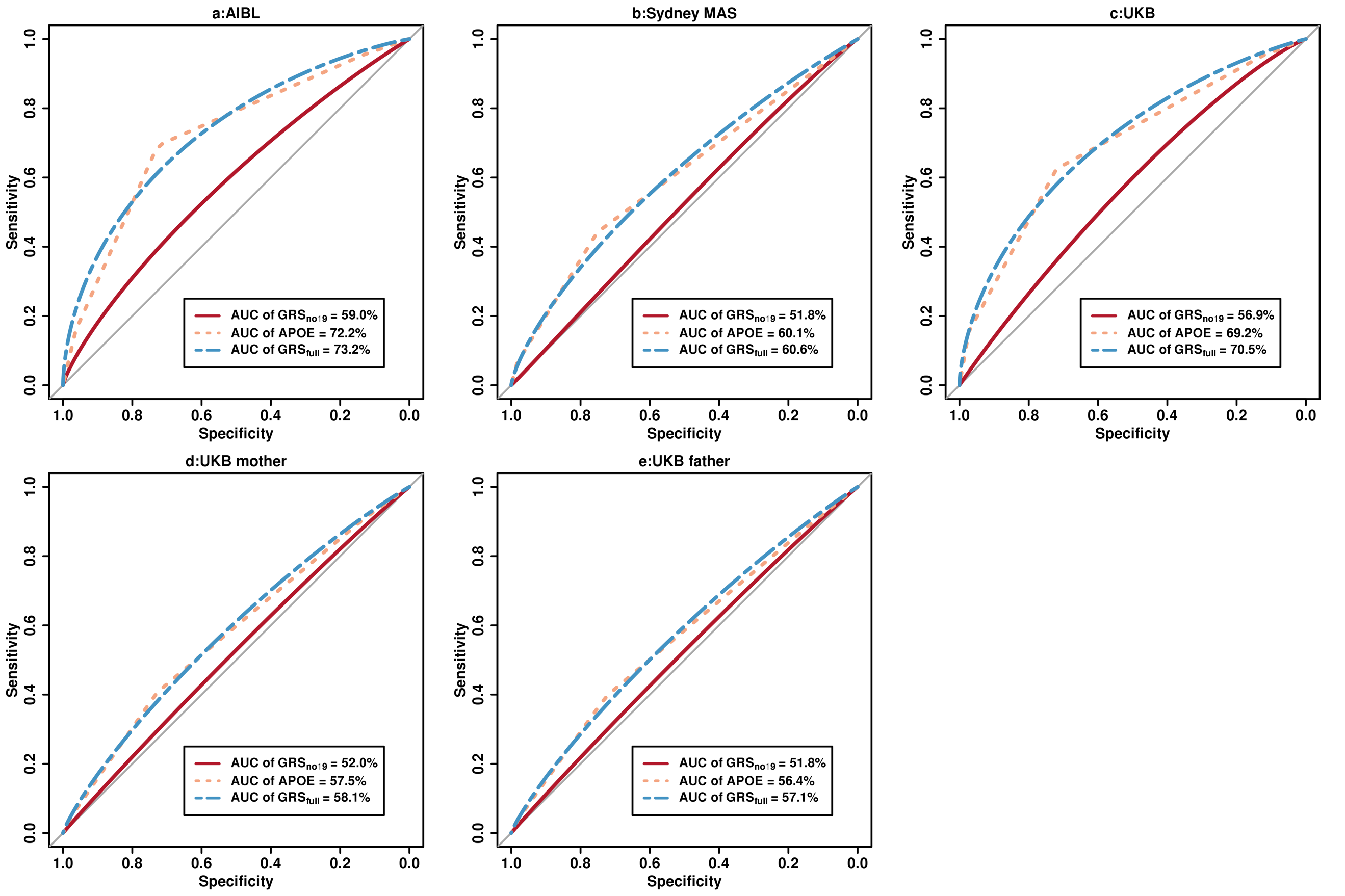


**Supplementary Figure 10**: Comparison of prediction performance (Area under the curve, AUC) between GRS and *APOE* in samples from AIBL(a), Sydney MAS(b), UKB(c), UKB mother(d) and UKB father(e).


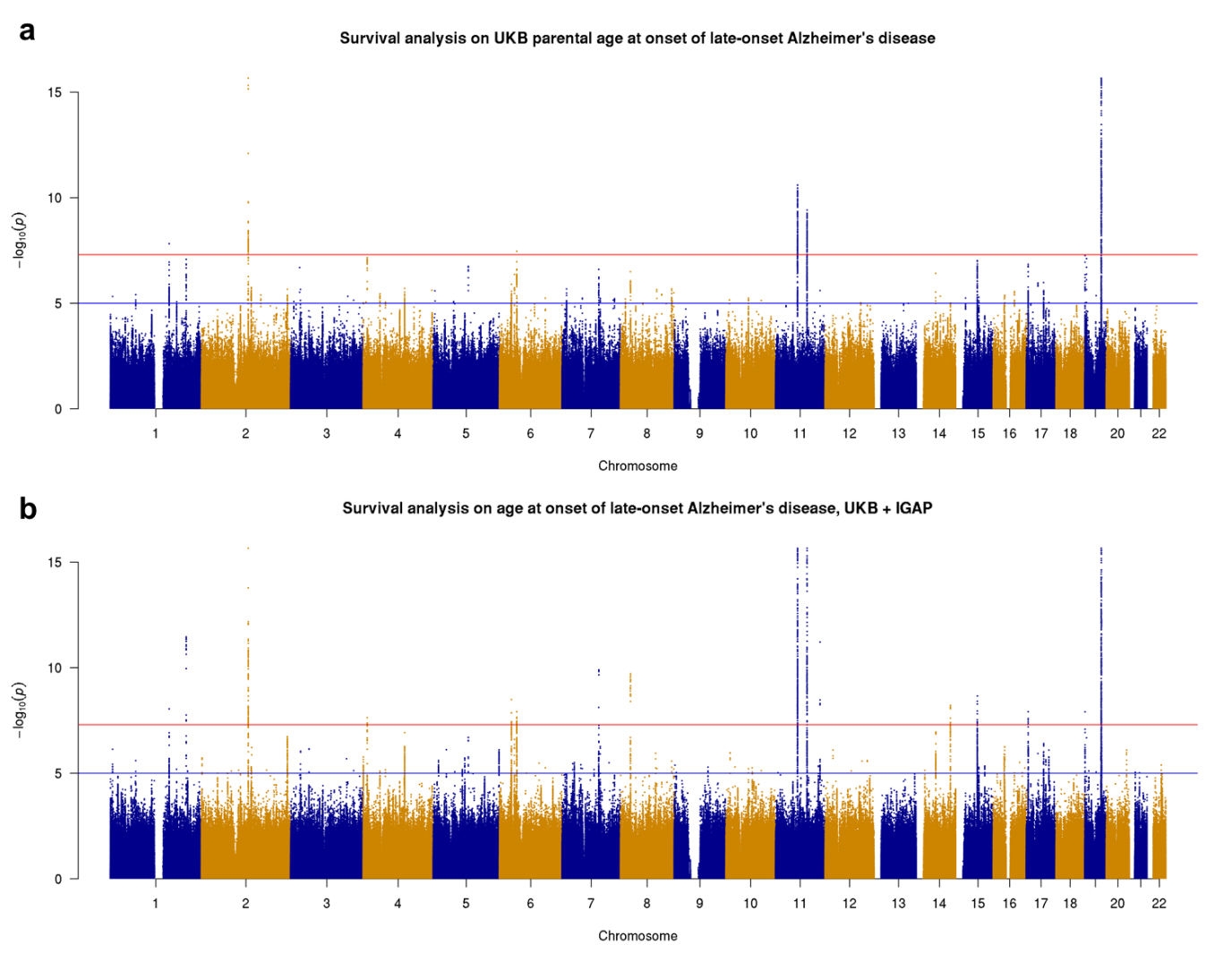


**Supplementary Figure 11**: Manhattan plot of the results from genome-wide survival analysis on age at onset of LOAD. y-axis (P-value) was truncated at 2.2x10^-16^. Red line represents 5x10^-8^, and blue line represents 1x10^-5^.


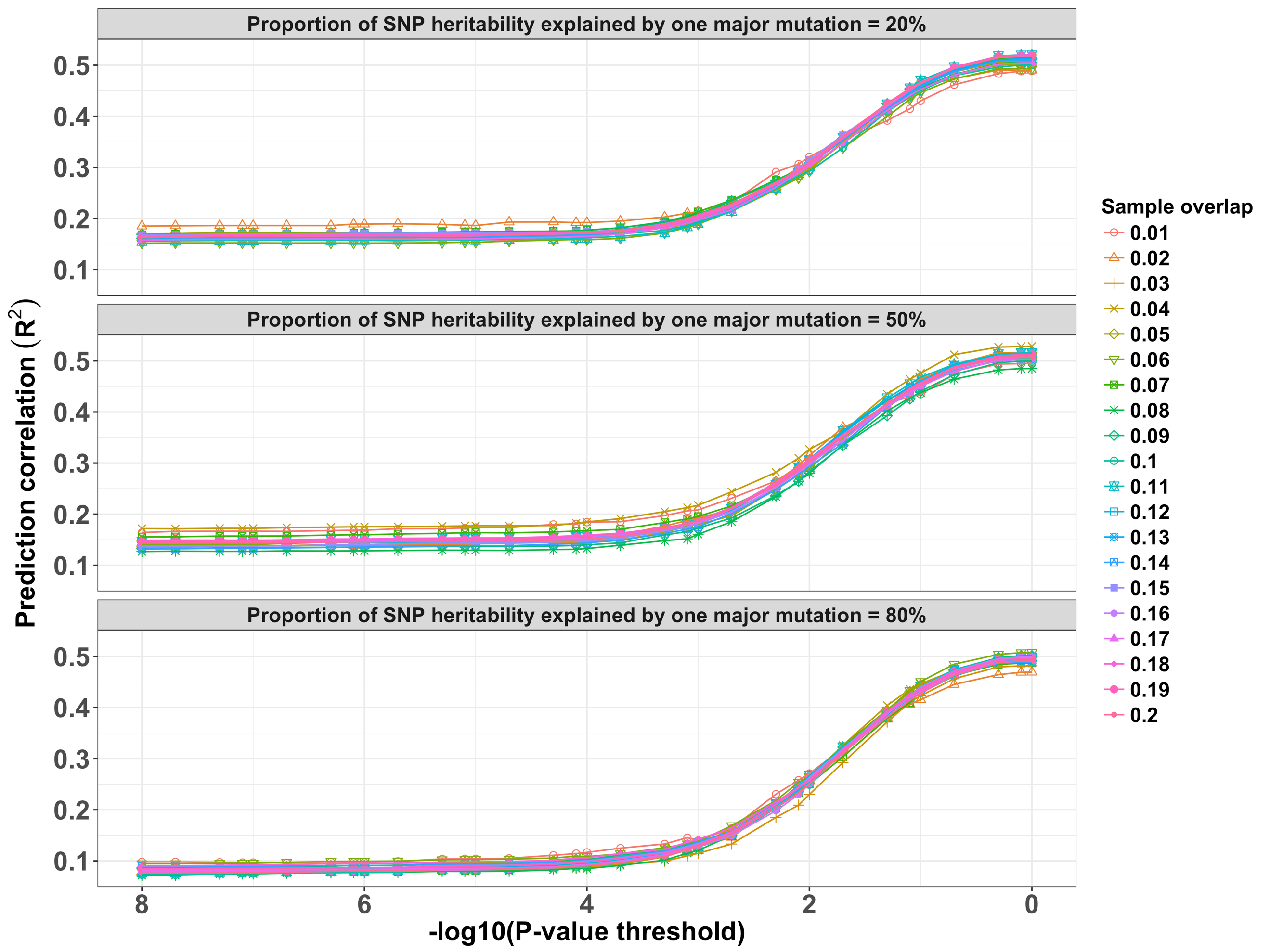


**Supplementary Figure 12**: The prediction pattern of GRS when the test set is included in the training set. We simulated a trait based on randomly selected 128 causal SNPs with heritability 0.2. One of the causal SNPs was set to explain 20%, 50% or 80% of the heritability. We randomly selected 90,000 individuals as training sets and chose a proportion of individuals from the training dataset (fraction ranges from 1% to 20%) as a test set. We performed GWAS on the training set and examined the performance of genetic risk score (based on LD clumping with different P-value thresholds) on the test set.


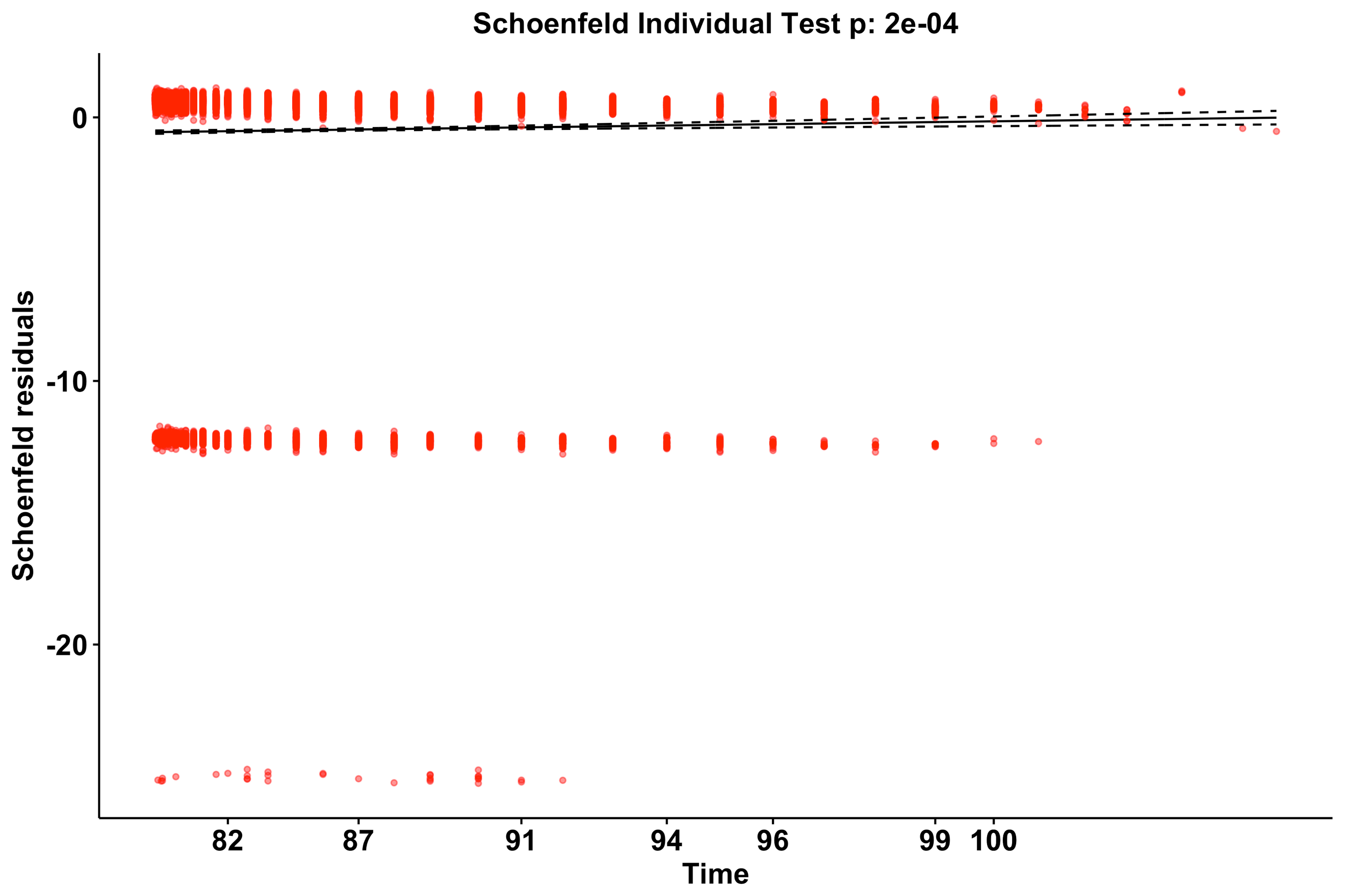


**Supplementary Figure 13**: The relationship between Schoenfeld residuals (rs1081105) and age. The black solid line is the linear regression line, and black dash line is the standard error.

**Supplementary Methods**

**Meta-analysis based on z-statistics: quantitative trait**

Assuming that the underlying model of the association study from which we have summary statistics is of the form

$y=bx+e$ (1)

where $b$ is the regression coefficient and x the genotype. The correlation between *x* and *y* is $R=b\sigma_{x}/\sigma_{y}$, with $\sigma_{x}$ and $\sigma_{y}$ the standard deviation of *x* and *y*. Let $\hat{b}$ and $\hat{R}$ be the estimates of *b* and *R*, respectively, *z* the z-score test statistic and *n* the sample size. Since:

$z^{2}=\frac{n\hat{R}^{2}}{1-\hat{R}^{2}} or \hat{R}^{2}=\frac{z^{2}}{n+z^{2}}$ (2)

then

$\mathrm{var}\left( \hat{b} \right)=\frac{\mathrm{var}\left( e \right)}{n\mathrm{var}\left( x \right)}=\frac{\mathrm{var}\left( y \right)-\hat{b}^{2}\mathrm{var}\left( x \right)}{n\mathrm{var}\left( x \right)}=\frac{\mathrm{var}\left( y \right)(1-\hat{R}^{2})}{n\mathrm{var}\left( x \right)}=\frac{\mathrm{var}\left( y \right)}{(n{+z}^{2})\mathrm{var}\left( x \right)}, and \mathrm{var}\left( \hat{R} \right)=\mathrm{var}\left( \hat{b} \right)\frac{\mathrm{var}\left( x \right)}{\mathrm{var}\left( y \right)}=\frac{1}{n{+z}^{2}}$ (3)

If we have two independent studies and no sample overlap, with $n_{i}$, $z_{i}$ and $\hat{R}_{i}$ for each study, assuming ${E(\hat{R}}_{i})$ is the same across studies, we can meta-analysis $\hat{R}_{i}$ using the fixed effect meta method:

$\hat{R}_{\mathrm{meta}}=\frac{\frac{\hat{R}_{1}}{\mathrm{var}(\hat{R}_{1})}+\frac{\hat{R}_{2}}{\mathrm{var}(\hat{R}_{2})}}{\frac{1}{\mathrm{var}(\hat{R}_{1})}+\frac{1}{\mathrm{var}(\hat{R}_{2})}}and \mathrm{var}\left( \hat{R}_{\mathrm{meta}} \right)=\frac{1}{\frac{1}{\mathrm{var}(\hat{R}_{1})}+\frac{1}{\mathrm{var}(\hat{R}_{2})}}$ (4)

Therefore,

$z_{\mathrm{meta}}=\frac{\hat{R}_{\mathrm{meta}}}{\sqrt{var\left( \hat{R}_{\mathrm{meta}} \right)}}=\frac{z_{1}\sqrt{n_{1}+z_{1}^{2}}+z_{2}\sqrt{n_{2}+z_{2}^{2}}}{\sqrt{n_{1}+z_{1}^{2}+n_{2}+z_{2}^{2}}}$ (5)

If $z^{2}\ll n$ (which is true is most cases),

$z_{\mathrm{meta}}\approx\frac{z_{1}\sqrt{n_{1}}+z_{2}\sqrt{n_{2}}}{\sqrt{n_{1}+n_{2}}}$ (6)

Therefore, the meta-analysis can be performed from the z-score test statistics and sample size from each study.

Now we consider the case where the phenotype in one of the studies is the sum of paternal (father) *y*_f_ and maternal (mother) *y*_m_ phenotypes, and the association study is performed using the summed parental phenotype ($y_{m+f}=y_{m}+y_{f}$) of an individual with their genotype.

$y_{m+f}=y_{m}+y_{f}=b_{m+f}x+e and \hat{b}_{m+f}=\frac{\mathrm{cov}(y_{m}+y_{f},x)}{\mathrm{var}(x)}=\frac{\mathrm{cov}(y_{m},x)}{\mathrm{var}(x)}+\frac{\mathrm{cov}(y_{f},x)}{\mathrm{var}(x)}$ (7)

Assuming that the genotype-phenotype association is the same in the parental and current generation, $\mathrm{cov}\left( y_{m},x \right)=\mathrm{cov}\left( y_{f},x \right)=½\mathrm{cov}(y,x)$. Therefore, $E\left( \hat{b}_{m+f} \right)=\frac{1}{2}b+\frac{1}{2}b=b$, where $b$ is the SNP effect on the phenotype when they are from the same individual.

The expectation of estimated correlation $\hat{R}_{m+f}$ between parental phenotype ($y_{m+f}$) and the offspring genotype is:

${E(\hat{R}}_{m+f})={E(\hat{b}}_{m+f})\frac{\sigma_{x}}{\sqrt{\mathrm{var}(y_{m+f})}}=b\frac{\sigma_{x}}{\sqrt{2\mathrm{var}(y)}}=\frac{b\sigma_{x}}{\sqrt{2}\sigma_{y}}=\frac{R}{\sqrt{2}}$ (8)

$\mathrm{var}\left( \hat{R}_{m+f} \right)=\frac{1}{n{+z}^{2}}$ (9)

Where *R* is the expectation of correlation between phenotype and genotype from the same individual as defined previously. Hence, for a proxy-phenotype association study using the summed parental phenotype, the expected regression coefficient ($b_{m+f}$) is the same as for an association study with individual’s own phenotype (*b*), but the expected correlation coefficient is reduced by $\sqrt{2}$.

We now consider the case where study 1 has the individual phenotype and in study 2 the sum of paternal (father) *y*_f_ and maternal (mother) *y*_m_ phenotype is used. To meta-analysis $\hat{R}$ from two studies, we need to multiply $\hat{R}_{m+f}$from study 2 by $\sqrt{2}$ so that it is in the same scale of $\hat{R}_{1}$ from study 1.

$\hat{R}_{\mathrm{meta}}=\frac{\frac{\hat{R}_{1}}{\mathrm{var}(\hat{R}_{1})}+\frac{\sqrt{2}\hat{R}_{m+f}}{2\mathrm{var}(\hat{R}_{m+f})}}{\frac{1}{\mathrm{var}(\hat{R}_{1})}+\frac{1}{2\mathrm{var}(\hat{R}_{m+f})}}and \mathrm{var}\left( \hat{R}_{\mathrm{meta}} \right)=\frac{1}{\frac{1}{\mathrm{var}(\hat{R}_{1})}+\frac{1}{2\mathrm{var}(\hat{R}_{m+f})}}$ (10)

$z_{\mathrm{meta}}=\frac{\hat{R}_{\mathrm{meta}}}{\sqrt{\mathrm{var}\left( \hat{R}_{\mathrm{meta}} \right)}}=\frac{z_{1}\sqrt{n_{1}+z_{1}^{2}}+z_{2}\sqrt{\frac{n_{2}+z_{2}^{2}}{2}}}{\sqrt{n_{1}+z_{1}^{2}+\frac{n_{2}+z_{2}^{2}}{2}}}\approx\frac{z_{1}\sqrt{n_{1}}+z_{2}\sqrt{\frac{n_{2}}{2}}}{\sqrt{n_{1}+\frac{n_{2}}{2}}}$ (11)

Therefore, the sample size used in the meta-analysis using the z-statistics should be divided by two for the summary statistics based on parental phenotype (paternal phenotype + maternal phenotype). The loss of information by using the parental phenotypes leads to its effective sample size being $\frac{n_{2}}{2}$.

**Meta-analysis based on z-statistics: binary trait**

When *y* is a binary trait (e.g., case and control), logistic regression would be used to estimate the effect size of *x*. The effect size $b_{\mathrm{logistic}}$ is log(odds ratio) and it is independent of disease ascertainment. The variance of estimated $\hat{b}_{\mathrm{logistic}}$ is, approximately ^16^:

$var(\hat{b}_{\mathrm{logistic}})\approx\frac{\mathrm{var}(y)}{n\mathrm{var}(x)}\approx\frac{1}{2n\mathrm{var}(x)P(1-P)}$ (12)

where *P* is the proportion of cases in the study and *n* is the total sample size of case and control samples. Therefore, the correlation *R* between *y* and *x* can be estimated as:

$\hat{R}=\frac{z}{\sqrt{n+z^{2}}} =\frac{{\hat{b}_{\mathrm{logistic}}}/{\sqrt{\mathrm{var}(\hat{b}_{\mathrm{logistic}})}}}{\sqrt{n+{\hat{b}_{\mathrm{logistic}}}^{2}/\mathrm{var}(\hat{b}_{\mathrm{logistic}})}}=\frac{\hat{b}_{\mathrm{logistic}}}{\sqrt{n\mathrm{var}(\hat{b}_{\mathrm{logistic}})+{\hat{b}_{\mathrm{logistic}}}^{2}}}\approx\frac{\hat{b}_{\mathrm{logistic}}}{\sqrt{n\mathrm{var}(\hat{b}_{\mathrm{logistic}})}}\approx\hat{b}_{\mathrm{logistic}}\sqrt{2\mathrm{var}(x)P(1-P)}$ (13)

$\mathrm{var}\left( \hat{R} \right)=\frac{1}{n{+z}^{2}}\approx\frac{1}{n}$ (14)

Since $E(\hat{b}_{\mathrm{logistic}})$ is usually assumed to be same across studies, $R$ in each study depends on the proportion of cases (*P*). To derive the correct weights for a meta-analysis based upon z-statistics, we use correlation estimates that are transformed to a scale ($R^{'}$) where they have the same expectations across studies:

$E(\hat{R}^{'})=\frac{E\left( \hat{R} \right)}{\sqrt{P\left( 1-P \right)}}\approx{E(\hat{b}}_{\mathrm{logistic}})\sqrt{2\mathrm{var}\left( x \right)} and \mathrm{var}\left( \hat{R}^{'} \right)\approx\frac{1}{P(1-P)n}$ (15)

Therefore, $z_{\mathrm{meta}}$ can be calculated based on the fixed effect meta method:

$z_{\mathrm{meta}}=\frac{{\hat{R}'}_{\mathrm{meta}}}{\sqrt{\mathrm{var}\left( {\hat{R}'}_{\mathrm{meta}} \right)}}=\frac{\frac{\hat{R}_{1}^{'}}{\mathrm{var}(\hat{R}_{1}^{'})}+\frac{\hat{R}_{2}^{'}}{\mathrm{var}(\hat{R}_{2}^{'})}}{\sqrt{\frac{1}{\mathrm{var}(\hat{R}_{1}^{'})}+\frac{1}{\mathrm{var}(\hat{R}_{2}^{'})}}}\approx\frac{\hat{R}_{1}^{'}n_{1}P_{1}\left( 1-P_{1} \right)+\hat{R}_{2}^{'}n_{2}P_{2}\left( 1-P_{2} \right)}{\sqrt{n_{1}P_{1}\left( 1-P_{1} \right)+n_{2}P_{2}\left( 1-P_{2} \right)}}=\frac{\hat{R}_{1}n_{1}\sqrt{P_{1}\left( 1-P_{1} \right)}+\hat{R}_{2}n_{2}\sqrt{P_{2}\left( 1-P_{2} \right)}}{\sqrt{n_{1}P_{1}\left( 1-P_{1} \right)+n_{2}P_{2}\left( 1-P_{2} \right)}}\approx\frac{z_{1}\sqrt{{n_{1}P}_{1}\left( 1-P_{1} \right)}+z_{2}\sqrt{n_{2}P_{2}\left( 1-P_{2} \right)}}{\sqrt{n_{1}P_{1}\left( 1-P_{1} \right)+n_{2}P_{2}\left( 1-P_{2} \right)}}$ (16)

As described in Liu et al ^11^, the effective sample size (sample size under balanced design) can be defined as $n_{\mathrm{eff}}=4nP(1-P)$, $z_{\mathrm{meta}}$ can then be re-written as:

$z_{\mathrm{meta}}\approx\frac{z_{1}\sqrt{n_{eff1}}+z_{2}\sqrt{n_{eff2}}}{\sqrt{n_{eff1}+n_{eff2}}}$ (17)

If the second GWAS summary is based on proxy samples ($y_{m+f}=y_{m}+y_{f}$). Assuming $y_{m}$ and $y_{f}$ are independent, and that their proportion of cases $P_{m}$ and $P_{f}$ are similar $P_{m}\approx P_{f}\approx\bar{P}$, then:

${E(\hat{R}}_{m+f})=\frac{\mathrm{cov}(y_{m+f},x)}{\sqrt{\mathrm{var}(y_{m+f})\mathrm{var}(x)}}=\frac{\mathrm{cov}\left( y_{m},x \right)+\mathrm{cov}(y_{f},x)}{\sqrt{\mathrm{var}(y_{m+f})\mathrm{var}(x)}}\approx\frac{R}{\sqrt{2}}$ (18)

Similar with quantitative trait under this condition, $z_{\mathrm{meta}}$ can then be defined as:

$z_{\mathrm{meta}}\approx\frac{z_{1}\sqrt{n_{1}P_{1}(1-P_{1})}+z_{2}\sqrt{\frac{n_{2}\bar{P}(1-\bar{P})}{2}}}{\sqrt{n_{1}P_{1}(1-P_{1})+\frac{n_{2}\bar{P}(1-\bar{P})}{2}}}$ (19)

When phenotype is the sum of paternal and maternal phenotype (Jansen et al.^3^ used this phenotype), the effective sample size$n_{\mathrm{eff}}=n_{m\_eff}+n_{f\_eff}=P_{m}\left( 1-P_{m} \right)N+P_{f}\left( 1-P_{f} \right)N=\left( P_{m}+P_{f}-P_{m}^{2}-P_{f}^{2} \right)N=\{P_{m}+P_{f}-\frac{1}{2}\left( P_{m}+P_{f} \right)^{2}-\frac{1}{2}\left( P_{m}-P_{f} \right)^{2}\}N\approx\left\{ P_{m}+P_{f}-\frac{1}{2}\left( P_{m}+P_{f} \right)^{2} \right\}N=\left( P_{m}+P_{f} \right)\left( 1-\frac{1}{2}\left( P_{m}+P_{f} \right) \right)N=2\bar{P}\left( 1-\bar{P} \right)N$. Therefore, $z_{\mathrm{meta}}$ can still be re-written as:

$z_{\mathrm{meta}}\approx\frac{z_{1}\sqrt{n_{eff1}}+z_{2}\sqrt{n_{eff2}}}{\sqrt{n_{eff1}+n_{eff2}}}$ (20)

**Reference**

1. Lambert, J.C. *et al.* Meta-analysis of 74,046 individuals identifies 11 new susceptibility loci for Alzheimer's disease. *Nat Genet* **45**, 1452-1458 (2013).

2. Marioni, R.E. *et al.* GWAS on family history of Alzheimer's disease. *Transl Psychiatry* **8**, 99 (2018).

3. Jansen, I.E. *et al.* Genome-wide meta-analysis identifies new loci and functional pathways influencing Alzheimer's disease risk. *Nat Genet* **51**, 404-413 (2019).

4. McLaren, W. *et al.* The Ensembl Variant Effect Predictor. *Genome Biol* **17**, 122 (2016).

5. Lee, S.H. *et al.* Estimation and partitioning of polygenic variation captured by common SNPs for Alzheimer's disease, multiple sclerosis and endometriosis. *Hum Mol Genet* **22**, 832-841 (2013).

6. Lee, S.H., Wray, N.R., Goddard, M.E. & Visscher, P.M. Estimating missing heritability for disease from genome-wide association studies. *Am J Hum Genet* **88**, 294-305 (2011).

7. Ridge, P.G., Mukherjee, S., Crane, P.K., Kauwe, J.S. & Alzheimer's Disease Genetics, C. Alzheimer's disease: analyzing the missing heritability. *PLoS One* **8**, e79771 (2013).

8. Brainstorm, C. *et al.* Analysis of shared heritability in common disorders of the brain. *Science* **360**(2018).

9. Bulik-Sullivan, B.K. *et al.* LD Score regression distinguishes confounding from polygenicity in genome-wide association studies. *Nat Genet* **47**, 291-295 (2015).

10. Harold, D. *et al.* Erratum: Genome-wide association study identifies variants at CLU and PICALM associated with Alzheimer's disease. *Nature Genetics* **41**, 1156-1156 (2009).

11. Liu, J.Z., Erlich, Y. & Pickrell, J.K. Case-control association mapping by proxy using family history of disease. *Nat Genet* **49**, 325-331 (2017).

12. Kunkle, B.W. *et al.* Genetic meta-analysis of diagnosed Alzheimer's disease identifies new risk loci and implicates Abeta, tau, immunity and lipid processing. *Nat Genet* **51**, 414-430 (2019).

13. Hollingworth, P. *et al.* Common variants at ABCA7, MS4A6A/MS4A4E, EPHA1, CD33 and CD2AP are associated with Alzheimer's disease. *Nat Genet* **43**, 429-435 (2011).

14. Naj, A.C. *et al.* Common variants at MS4A4/MS4A6E, CD2AP, CD33 and EPHA1 are associated with late-onset Alzheimer's disease. *Nat Genet* **43**, 436-441 (2011).

15. Ripley, A.C.a.B.D. boot: Bootstrap R (S-Plus) Functions. (2020).

16. Fahrmeir, L. & Kaufmann, H. Consistency and Asymptotic Normality of the Maximum Likelihood Estimator in Generalized Linear Models. *The Annals of Statistics* **13**, 342-368 (1985).
