# Supplementary material for: Risk prediction of late-onset Alzheimer’s disease implies an oligogenic architecture
Source: Nat Commun. 2020 Sep 23;11:4799. doi: 10.1038/s41467-020-18534-1 (PMC7511365; doi:10.1038/s41467-020-18534-1)
Supplement: Supplementary file 2 — Peer Review File [file 41467_2020_18534_MOESM2_ESM.docx]

Peer Review Information

**Journal:** Nature Communications

**Manuscript title:** Risk prediction of late-onset Alzheimer’s disease implies an oligogenic architecture

**Corresponding author name(s):** Prof Peter Visscher

**Editorial notes:**

**Transferred manuscripts**

This manuscript has been previously reviewed at another journal that is not operating a transparent peer review scheme. This document only contains reviewer comments and rebuttal letters for versions considered at *Nature Communications*.

**Reviewer comments & decisions:**

**Reviewer comments, first version:**

Reviewer #2 (Remarks to the Author):

i remain ambivalent to this ms.

The dementia cases will include FTD, DLB and Vascular dementia as well as AD. Even AIBL will include mixed and DlB cases. The sample sets are a mix of small datasets with good diagnoses (but by no means perfect) and large datasets with almost no diagnosis and the title seeks to distinguish between oligo and poly!!! i note the entry criteria for AIBL indirectly include a family history and wonder whether that will bias analysis

Since the submission of the paper other new loci have been reported. I am therefore not really sure what is the point of the distinction between "oligo" and "poly" is in the context of already 50 loci. I would strongly suspect that as analysis increases, we will find more and more loci with lower and lower odds ratios. At what point one says "enough already" will be a matter almost of semantics and not science


Reviewer #4 (Remarks to the Author):

Zhang and colleagues present a very interesting analysis of the genetic architecture of late-onset Alzheimer’s disease. Using summary statistics from multiple GWAS, they find that only a small proportion of SNPs are likely to contribute to the heritability of LOAD. This architecture clearly differs from the architecture of metabolic traits for which genome wide association studies have been performed. These results are also consistent with the much greater p-value threshold which has been found to provide ideal predictive power for polygenic scores other traits and disease (e.g in Khera et al 2018 Nature Genetics, the proportion of causal SNPs that generated the most strongly associated polygenic score was found to vary from 0.1 to 10% versus the 0.01% reported in the current study).

The consistent results reported using different summary statistics, pruning thresholds and LD-PRED is convincing. I have no comments after what was covered in the first revision and recommend acceptance.

**Author rebuttal, first version:**

Reviewer #2 (Remarks to the Author):

I remain ambivalent to this ms.

The dementia cases will include FTD, DLB and Vascular dementia as well as AD. Even AIBL will include mixed and DlB cases. The sample sets are a mix of small datasets with good diagnoses (but by no means perfect) and large datasets with almost no diagnosis and the title seeks to distinguish between oligo and poly!!! i note the entry criteria for AIBL indirectly include a family history and wonder whether that will bias analysis.

*We agree with the reviewer that dementia cases could be heterogeneous in terms of clinical (phenotypic) diagnosis. We have declared the potential limitation of heterogeneity on the estimation of the number of causal SNPs (Page 10, Line 21). We note that although the datasets with real cases are small, the prediction pattern of genetic risk score (GRS) is consistent across these datasets. The pattern suggests the prediction performance of GRS increases with the use of a more stringent P-value threshold for SNP selection and therefore with fewer SNPs in the model. This result is similar to that from direct estimation based on the summary statistics, which shows that the number of non-zero effects on LOAD is around 100. All these results on the genetics suggest our estimation is robust. For the dataset (UK Biobank) with parental cases (which is reported), the genetic correlation between GWAS based on parental cases/controls and GWAS from Lambert et al., 2013, Nature genetics (real cases/controls) is not significantly different from unity (rG = 0.92, s.e. = 0.15, Supplementary Table 1). A genetic correlation of 1 implies that the traits are genetically the same, but does not preclude having more measurement error in the non-clinically defined cases (and controls). The high genetic correlation shows that there is no significant difference when treating LOAD parental cases/controls from UK Biobank as real cases/controls and, therefore, we think it is appropriate to use parental cases/controls in our study.*

*Regarding the samples from the Australian Imaging Biomarkers and Lifestyle Study (AIBL), although they are likely to have self-selection bias towards those with a family history of dementia, the proportion of APOE ε4 carriers in this cohort were consistent with previous estimates of the Australian population (Ellis et al., 2009, International Psychogeriatrics), suggesting there is no bias on the criteria or the effect of the bias is small. Therefore, we think the potential bias in sample selection will not affect our analysis.*

Since the submission of the paper other new loci have been reported. I am therefore not really sure what is the point of the distinction between "oligo" and "poly" is in the context of already 50 loci. I would strongly suspect that as analysis increases, we will find more and more loci with lower and lower odds ratios. At what point one says "enough already" will be a matter almost of semantics and not science.

*We thank the reviewer for this comment and agree that the referee makes a valid point about what counts as ‘oligo’ versus ‘poly’. We claimed late-onset Alzheimer's disease (LOAD) as an oligogenic disease since only 0.01% of SNPs with MAF>1% have non-zero effects on LOAD, which contrasts with other brain-related polygenic diseases. For example, the proportion of HapMap3 SNPs with non-zero effects sizes on schizophrenia, major depression and Parkinson’s disease was estimated to be 17.5% (s.e. = 2.4%), 3.2% (s.e. = 0.8%) and 16.4% (s.e. = 5.8%), respectively. The huge difference in this proportion suggests LOAD having a substantially different genetic architecture to other disorders of the brain. Such difference in genetic architecture implies that the function and mechanism of genetic perturbations in LOAD may be more tractable than in, say, schizophrenia. It also implies a relatively faster rate of diminishing returns from ever-larger common variant GWAS in LOAD versus other brain-related disorders.*

*We agree with the reviewer that more loci could be discovered in the future since the currently identified loci (~40) is still smaller than the estimated number (~100) in the manuscript. SNPs with small effects on LOAD can be detected while increasing the sample size. Besides, it should be noted that only common SNPs were considered in this study (1,056,154 HapMap3 SNPs and two APOE SNPs: rs429358 and rs7412), rare variants were not taken into account and larger samples are required to discover these variants.*

Reviewer #4 has no comments.

**Reviewer comments, second version:**

**Author rebuttal, second version:**

**Reviewer comments, third version:**

**Author rebuttal, third version:**
